# Mobile Health–Supported HIV Self-Testing Strategy Among Urban Refugee and Displaced Youth in Kampala, Uganda: Protocol for a Cluster Randomized Trial (Tushirikiane, Supporting Each Other)

**DOI:** 10.2196/26192

**Published:** 2021-02-02

**Authors:** Carmen Logie, Moses Okumu, Robert Hakiza, Daniel Kibuuka Musoke, Isha Berry, Simon Mwima, Peter Kyambadde, Uwase Mimy Kiera, Miranda Loutet, Stella Neema, Katie Newby, Clara McNamee, Stefan D Baral, Richard Lester, Joshua Musinguzi, Lawrence Mbuagbaw

**Affiliations:** 1 Factor-Inwentash Faculty of Social Work University of Toronto Toronto, ON Canada; 2 School of Social Work University of North Carolina Chapel Hill Chapel Hill, NC United States; 3 Young African Refugees for Integral Development Kampala Uganda; 4 International Research Consortium Kampala Uganda; 5 Dalla Lana School of Public Health University of Toronto Toronto, ON Canada; 6 Uganda Ministry of Health Kampala Uganda; 7 Most At Risk Population Initiative Mulago Hospital Kampala Uganda; 8 Bukedi Prevention Institute Kampala Uganda; 9 Department of Sociology and Anthropology Makerere University Kampala Uganda; 10 Centre for Research in Psychology and Sport Sciences School of Life and Medical Sciences University of Hertfordshire Hertfordshire United Kingdom; 11 Johns Hopkins Bloomberg School of Public Health Johns Hopkins University Baltimore, MD United States; 12 Department of Medicine University of British Columbia Vancouver, BC Canada; 13 Department of Health Research Methods, Evidence and Impact McMaster University Hamilton, ON Canada

**Keywords:** adolescents and youth, implementation research, HIV testing, mobile health, refugee, Uganda

## Abstract

**Background:**

HIV is the leading cause of mortality among youth in sub-Saharan Africa. Uganda hosts over 1.43 million refugees, and more than 83,000 live in Kampala, largely in informal settlements. There is limited information about HIV testing uptake and preferences among urban refugee and displaced youth. HIV self-testing is a promising method for increasing testing uptake. Further, mobile health (mHealth) interventions have been effective in increasing HIV testing uptake and could be particularly useful among youth.

**Objective:**

This study aims to evaluate the feasibility and effectiveness of two HIV self-testing implementation strategies (HIV self-testing intervention alone and HIV self-testing combined with an mHealth intervention) in comparison with the HIV testing standard of care in terms of HIV testing outcomes among refugee/displaced youth aged 16 to 24 years in Kampala, Uganda.

**Methods:**

A three-arm cluster randomized controlled trial will be implemented across five informal settlements grouped into three sites, based on proximity, and randomization will be performed with a 1:1:1 method. Approximately 450 adolescents (150 per cluster) will be enrolled and followed for 12 months. Data will be collected at the following three time points: baseline enrollment, 8 months after enrollment, and 12 months after enrollment. Primary outcomes (HIV testing frequency, HIV status knowledge, linkage to confirmatory testing, and linkage to HIV care) and secondary outcomes (depression, condom use efficacy, consistent condom use, sexual relationship power, HIV stigma, and adolescent sexual and reproductive health stigma) will be evaluated.

**Results:**

The study has been conducted in accordance with CONSORT (Consolidated Standards of Reporting Trials) guidelines. The study has received ethical approval from the University of Toronto (June 14, 2019), Mildmay Uganda (November 11, 2019), and the Uganda National Council for Science and Technology (August 3, 2020). The Tushirikiane trial launched in February 2020, recruiting a total of 452 participants. Data collection was paused for 8 months due to COVID-19. Data collection for wave 2 resumed in November 2020, and as of December 10, 2020, a total of 295 participants have been followed-up. The third, and final, wave of data collection will be conducted between February and March 2021.

**Conclusions:**

This study will contribute to the knowledge of differentiated HIV testing implementation strategies for urban refugee and displaced youth living in informal settlements. We will share the findings in peer-reviewed manuscripts and conference presentations.

**Trial Registration:**

ClinicalTrials.gov NCT04504097; https://clinicaltrials.gov/ct2/show/NCT04504097.

**International Registered Report Identifier (IRRID):**

DERR1-10.2196/26192

## Introduction

HIV is the leading cause of death among young people globally and the main cause of death among adolescents and young people in sub-Saharan Africa [1]. In sub-Saharan Africa, half of newly infected adolescents and young people are girls [2,3]. Uganda’s 2018 HIV prevalence among adolescent girls was over double that of adolescent boys, with 14,000 young women aged 15 to 24 reporting new HIV infections compared with 5000 young men [4]. Only 53.6% of adolescent girls in Uganda know their HIV status, and this is far below the Joint United Nations Program on HIV/AIDS (UNAIDS) goal of 90% of persons living with HIV knowing their status by 2020 to achieve an AIDS-free generation [5,6]. HIV testing is a critical entry point for access to HIV prevention and control solutions, specifically access to antiretroviral therapy, which is vital for preventing onward HIV transmission [7,8].

There are more than 79.5 million forcibly displaced persons worldwide [9], and refugee and displaced persons are largely underserved by current HIV prevention strategies [10]. HIV vulnerabilities among displaced/refugee adolescents are shaped by a complex interplay of factors, including poverty, violence, host community HIV prevalence, urbanization, HIV testing and care access, and living conditions [10-12]. Uganda hosts more than 1.4 million refugees [13], with 62% aged below 18 years [14]. Kampala, Uganda’s capital city, hosts over 83,000 refugees [13], and 27% are aged 15 to 24 years [15]. It is important to explore HIV testing needs among urban refugees. There is a global trend of refugee urbanization, with refugees increasingly moving from refugee settlements to urban areas for employment and education opportunities [16-19]. Urban refugees and displaced adolescents and young people are at the nexus of HIV disparities among adolescents, displaced persons, and slum dwellers, and yet, there are knowledge gaps in the optimal HIV prevention for these groups [20,21].

HIV self-testing is a promising strategy to engage marginalized populations [20]. HIV self-testing can represent a convenient and private option, and may reduce stigma compared with clinic-based testing [21]. HIV self-testing has demonstrated acceptability, feasibility, and usability, with minimal harm, and is associated with increased HIV testing among individuals, including adolescents across Southern Africa and Malawi [20,22,23]. Mobile health (mHealth) strategies (digital media on mobile devices) have been efficacious in improving antiretroviral therapy adherence and HIV and sexually transmitted infection testing, and are relevant for the way youth learn and socialize [24-26]. In particular, two-way SMS-based mHealth interventions that are interactive and supportive have been found to be more efficacious in increasing adherence than one-way messages/reminders [27,28]. However, few studies have integrated mHealth into HIV self-testing delivery with adolescents or focused on optimal HIV self-testing delivery strategies with displaced/refugee youth.

Global HIV self-testing knowledge gaps include strategies to facilitate linkage to confirmatory testing and HIV care for persons testing positive [29]. Identifying strategies to promote linkage to HIV care is essential to realize the public health impact of HIV self-testing [30]. A recent systematic review reported a dearth of evidence-based strategies for linkage to HIV care following positive HIV self-testing results among adolescents [31]. This is an urgent knowledge gap that we aim to address with bidirectional text messaging strategies. We also aim to address the lack of evidence regarding the acceptability and feasibility of HIV self-testing among refugee and displaced persons [32]. In Uganda, HIV self-testing was feasible and acceptable; demonstrated the ability to identify new HIV diagnoses among men who have sex with men [33]; and was associated with increased recent and frequent testing among sex workers [34].

Tushirikiane, roughly translating to supporting each other in Swahili, aims to address these knowledge gaps among displaced and refugee adolescent youth in Uganda by testing a mHealth support strategy alongside HIV self-testing delivery to increase routine HIV testing uptake. The findings can be used to inform the implementation and scale-up of HIV self-testing programs with displaced and refugee adolescents across Uganda, sub-Saharan Africa, and other humanitarian contexts.

## Methods

### Study Aim and Objectives

This study aims to evaluate the feasibility and clinical effectiveness of two HIV self-testing delivery approaches (HIV self-testing intervention alone and HIV self-testing combined with a two-way supportive SMS mHealth intervention) in comparison with the standard of care in terms of HIV testing uptake among refugee and displaced youth aged 16 to 24 years in Kampala, Uganda. The specific objectives of this study are to determine the effectiveness of the interventions on the following criteria: (1) increased frequency of HIV testing; (2) increased knowledge of HIV status; (3) increased linkage to confirmatory testing (for those with an HIV positive self-test); and (4) increased linkage to HIV care (for persons testing positive for HIV in HIV self-testing and confirmatory testing). Secondary outcomes include (1) depression, (2) condom use self-efficacy, (3) consistent condom use, (4) sexual relationship power, (5) HIV stigma, and (6) adolescent sexual and reproductive health (SRH) stigma.

### Study Design

To evaluate the intervention effectiveness, we will conduct a cluster randomized controlled trial (cRCT), where informal settlements are randomized. The clusters include five informal settlements grouped into three sites that are randomized in a 1:1:1 method to one of three study arms. Although outcome data will be collected at the level of the individual, we selected cluster randomization over individual randomization because the intervention is implemented at the settlement level. A cluster randomized design addresses threats of internal validity. It reduces the possibility of experimental contamination due to the shared social and physical environments between youth in the same or nearby informal settlements [35]. Data will be collected at the following three time points: baseline enrollment into the intervention, 8 months after enrollment, and 12 months after enrollment.

### Study Setting

This trial is being conducted in Kampala, which is the capital of Uganda. Uganda’s progressive refugee policies provide refugees and displaced persons in refugee settlements a plot of land. Yet, with the 2006 Uganda Refugee Law, refugees forgo rights to humanitarian assistance if they leave these refugee settlements [16,17]. The convergence of this law with refugee urbanization contributes to extreme poverty among Kampala’s urban refugees, creating slum environments [16,36,37]. Among Ugandan youth aged 15 to 19 years, the HIV prevalence is estimated at 4% [38]. However, among vulnerable youth living in Kampala’s informal settlements, this is estimated at 37.2% [39]. The five informal settlements selected for this study are grouped into three sites based on their proximity to one another (Kabalagala/Kansanga, Katwe/Nsambya, and Rubaga) and have been purposively chosen because these communities host many displaced/refugee persons in Kampala [16,18,36,37]. We focused on these refugee communities, largely from the Democratic Republic of Congo, Rwanda, and Burundi [13], where refugees continue to arrive, owing to similarities in socioeconomic status and living conditions, health care access, and shared languages (French and Swahili).

### Study Population and Eligibility Criteria

A minimum of 432 youth (144 per cluster) between the ages of 16 and 24 years will be enrolled into this study and followed for 12 months. Individuals are eligible for inclusion if they meet the following criteria: (1) currently live in one of the following five informal settlements in Kampala: Kabalagala, Kansanga, Katwe, Nsambya, and Rubaga; (2) identify as a refugee/displaced person or have refugee/displaced parents; (3) are aged 16 to 24 years; (4) speak English, Swahili, Luganda, French, Kinyarwanda, or Kirundi; and (5) own a mobile phone. A brief eligibility screening (via phone, in person, or via WhatsApp) with interested participants will be conducted, asking self-reported HIV serostatus. Only participants reporting an HIV-negative status (baseline serostatus) will be eligible to participate.

### Participant Recruitment and Retention

The project team includes a refugee youth-focused community-based nongovernment organization that implements economic empowerment programs for refugee youth and holds expertise on youth engagement. The team also involves academics and practitioners from the Ministry of Health and HIV clinics. Additionally, this project engages peer navigators who identify as refugees or displaced persons (aged 18-24 years) to help with participant recruitment and to provide feedback on the study design and survey. Twelve peer navigators (six young women and six young men) recruited for this study have been identified by community-based collaborators, have experience working in the various study communities as health or peer educators, and are deeply respected and connected in their communities.

Participants will be recruited within each settlement using purposive methods, including word-of-mouth and venue-based sampling at refugee agencies and community events. Peer navigators will conduct peer-driven recruitment at each site, sharing youth-designed flyers for potential participants to contact (via email and mobile number) peer navigators to join the study. Community collaborators and peer navigators will facilitate participant retention. Specifically, peer navigators will use multiple study reminder strategies (eg, social media and texts) to maintain engagement. We will utilize existing outreach and services by local refugee agencies and community partners.

### Patient and Public Involvement in Research

Study collaborators at Young African Refugees for Integral Development (YARID) have been involved in the research from the initial stage of developing the research question and focus. We conducted a preliminary exploration of the needs and priorities of refugees and youth with YARID prior to developing this study, and those findings indicated a low prevalence of lifetime HIV testing, which reinforced the importance of this study. Peer navigators, who themselves are urban refugee youth living in Kampala’s informal settlements, provided feedback for the study design and outcomes; conducted recruitment and active engagement with study participants for retention; supported study implementation by facilitating linkages between participants and data collectors; pilot tested the survey to assess the time required to participate in the research; and will engage in multiple dissemination strategies for community members (eg, providing input for infographic design and sharing community reports with community stakeholders including the Ministry of Health).

### Intervention Description

**Figure 1:**
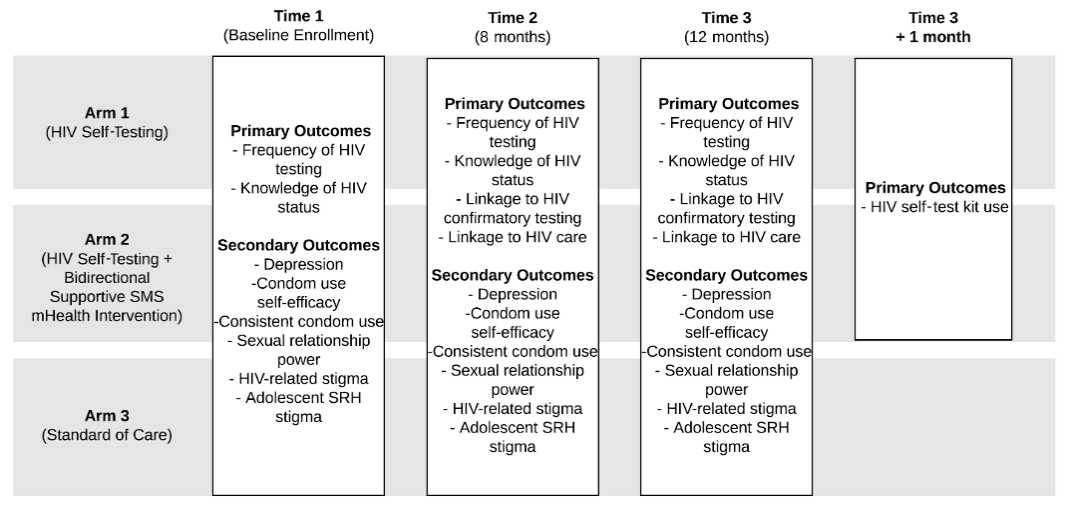
Study design for Tushirikiane, a cluster randomized trial of a mobile health (mHealth) HIV self-testing strategy among urban refugee and displaced youth in Kampala, Uganda. SRH: sexual and reproductive health.

The study has been designed as a three-arm cRCT consisting of two treatment arms and one control arm. Clusters will be randomized to one of the following three arms: (1) HIV self-testing, (2) HIV self-testing plus mHealth strategies (supportive text messages), and (3) standard of care (clinic-based HIV testing). The trial arms and interventions are described below and summarized in Figure 1.

#### Treatment Arm 1: HIV Self-Testing Intervention

Participants in this arm will be enrolled into the HIV self-testing intervention group and will receive HIV self-testing kits so they can perform HIV testing. At baseline enrollment, the peer navigator will provide an HIV self-testing kit (OraQuick Rapid HIV-1/2 Antibody Test, OraSure Technologies), which includes an oral swab test stick and tube solutions, a written detailed step-by-step description of how to correctly use the HIV self-testing kit, pictorial and written guides for the HIV self-testing kit, condoms and lubricant, information booklets on HIV and testing, and referral cards with the addresses and phone numbers of local clinics for confirmatory testing. The cards will also have the peer navigator’s phone number to allow participants to contact the peer navigator if they need additional information on how to use the kit or need support to go for confirmatory tests at local clinics. Instructions for the kit are in French, Luganda, Swahili, Kirundi, Kinyarwanda, and English and have been pilot tested for clarity and comprehensiveness with peer navigators. A 24-hour contact number will be provided to participants to text if/when they have questions. At follow-up visits, peer navigators will check in with participants about the HIV self-testing kit, distribute another HIV self-testing kit and condoms/lubricant, and screen for adverse events (eg, negative HIV self-testing–related experiences).

#### Treatment Arm 2: HIV Self-Testing Plus mHealth

Participants in this arm will be enrolled into the HIV self-testing group (as above) and on a web-based SMS platform hosted by WelTel [40,41]. WelTel is a nonprofit agency developing the mHealth intervention, in which participants receive weekly supportive bidirectional text messages asking how they are doing [42]. Participants are requested to reply “fine” or “not fine,” and those responding “not fine” will be contacted with support by a peer navigator. Participants in this arm will discuss the weekly WelTel messages with peer navigators and respond to the “not fine” messages within 2 days. If they do not reply to the initial SMS message within the specified timeframe, a peer navigator will follow up with them during that week. The WelTel system will manage the SMS intervention on a structured mobile phone platform (all SMS interactions are logged). WelTel’s two-way texting mHealth intervention may prompt participants to engage in HIV self-testing and/or to engage peer navigator support in decision making regarding HIV self-testing practices.

#### Arm 3: Standard of Care

Participants in this arm will be enrolled into a standard of care group. Participants will receive information about HIV testing, care, and support services at local clinics. They will be provided a pamphlet of information about HIV and HIV prevention strategies (written in French, English, Luganda, Kirundi, Kinyarwanda, and Swahili).

### Outcomes

#### Primary Outcomes

The primary outcomes measured in this trial are as follows:

1. Changes in HIV testing frequency: This is measured as participants’ self-reported last HIV test. To capture changes, this measure is assessed at all three study time points (baseline [Time 1], 8 months [Time 2], and 12 months [Time 3]).
2. Changes in knowledge of HIV status: To address social desirability bias challenges regarding self-reported HIV serostatus, multiple steps are employed. First, at each timepoint (Time 1, Time 2, and Time 3), participants are asked their current HIV status. At Time 3, participants are offered a completely voluntary rapid HIV test. Knowledge of HIV status will be assessed as correct for persons who agree to take the rapid test and correctly report their HIV status (prior to receiving the result).
3. Changes in linkage to confirmatory HIV testing: Participants in trial arms 1 and 2 (HIV self-testing and mHealth HIV self-testing intervention) are asked at Time 2 and Time 3 if they used their HIV self-testing kit. For those who respond affirmatively, they will be asked the result, and those who report a positive result will be asked if and where they received confirmatory testing.
4. Changes in linkage to HIV care: Participants who seroconvert during the study are asked the frequency of accessing HIV care service since diagnosis. This will be assessed at Time 2 and Time 3.
5. HIV self-testing kit use: To understand the frequency of HIV self-testing kit use and to reduce social desirability bias regarding HIV self-testing kit use, participants in trial arms 1 and 2 will be followed up 1 month after Time 3 to request for purchasing unused kits back. Participants will not be informed of this as an option prior to this time.

#### Secondary Outcomes

Secondary outcomes include changes in depression assessed using the Patient Health Questionnaire-9 items (PHQ-9) [43]; condom use self-efficacy measured with the Condom Use Efficacy Scale [44,45]; consistent condom use frequency assessed by asking participants if they used condoms every time (consistently) in the past 3 months; sexual relationship power assessed using the relationship control subscale from the Sexual and Reproductive Power Scale [46]; perceived HIV-related stigma with the perceived HIV-related stigma subscale of Steward et al [47]; and adolescent SRH stigma assessed with the Ugandan Adolescent SRH Stigma Scale [48] adapted from the Adolescent SRH scale by Hall et al [49].

### Sample Size and Power Analysis

Cluster sizes of 130 per group (n=390) are required to have 80% power (*P*<.05) to detect a 25% difference (39% [5] vs 64% tested) in HIV testing between any two groups from three pairwise comparisons (control vs arm 1, control vs arm 2, and arm 1 vs arm 2) for an odds ratio of 1.66. We assume an intraclass correlation coefficient of 0.013 based on HIV research on condom use in sub-Saharan Africa [50]. With 10% attrition, 432 participants (144 per cluster) are required. Computations were performed using RStudio version 3.3.0 (RStudio Team), based on formulae for multiple comparisons of proportions, and adjusted for design effect [51].

### Data Collection and Management

Outcome data will be collected at three time points as identified using a tablet-based survey application (QuickTapSurvey, Formstack for Time 1; SurveyCTO, Dobility for Time 2 and Time 3). Baseline data to characterize the study population include demographics and sexual history, which will be collected using these tools. Data will be automatically uploaded onto a secure password-protected server using an SSL certificate and will remain encrypted when not in use.

### Data Analysis Plan

Analysis and reporting will be conducted in accordance with CONSORT (Consolidated Standards of Reporting Trials) guidelines [52] (Multimedia Appendix 1). The analyst will be blinded to group allocation. A flow diagram will be used to illustrate patient flow (screening, randomization, allocation, and follow-up). Baseline data will be reported for all three arms and summarized as mean (SD) or median (first quartile, third quartile) for continuous variables and as count and number (percentage) for categorical variables. Primary analysis will involve intention-to-treat analysis (data from participants will be analyzed according to their allocation, irrespective of whether they actually received that intervention). Between-group comparisons will be performed using multilevel mixed effect logistic or linear regression models (to account for clustering), depending on which outcome is being evaluated. For these models, the intervention group will be entered as a fixed effect. The level of significance will be set at α=.05, but adjusted using the Bonferroni method for secondary outcomes [53]. The results will be expressed as odds ratios or mean differences as appropriate, accompanied by 95% CIs and *P* values. We will conduct adjusted analysis for the primary outcome (changes in HIV testing frequency) to investigate the role of various covariates in the relative effect. We will build mixed effect multilevel logistic regression models with the intervention group as the independent variable and HIV testing uptake in the past 3 months (yes/no) as the dependent variable. Covariates (eg, age) will be entered as a block. We will explore gender differences in primary and secondary intervention outcomes.

## Results

The Tushirikiane trial protocol has been approved by the Research Ethics Board of the University of Toronto (June 14, 2019), Mildmay Uganda Research Ethics Committee (November 11, 2019), and Uganda National Council for Science & Technology (August 3, 2020). The trial is registered at ClinicalTrials.gov (NCT04504097). The Tushirikiane trial launched on February 15, 2020, recruiting a total of 452 participants. Data collection was paused for 8 months owing to COVID-19. Data collection for wave 2 resumed on November 18, 2020, and as of December 10, 2020, a total of 295 participants have been followed up. Data collection for the third, and final, wave will be conducted between February and March 2021. The final follow-up to purchase back unused HIV self-testing kits will occur in June 2021.

## Discussion

### Study Implications

Although Uganda’s Ministry of Health currently recommends HIV self-testing, there are currently no national guidelines surrounding the optimal delivery of HIV self-testing for adolescents, young people, or refugees/displaced persons. This study is unique in Uganda and elsewhere [32] to outline a path to reducing the barriers to HIV testing faced by urban refugee and displaced youth living in slums and informal settlements. Refugee and displaced adolescents and youth are often not included in HIV and other SRH programs [54-56]. There is also a need for age and gender-disaggregated data to inform SRH programs with refugee and displaced youth, and our study will provide such data [57]. Our research has the potential to advance the HIV prevention and care cascades that involve the integration of social science, epidemiological, and health behavior theories in interventions to increase demand for (through HIV knowledge and peer support) and supply of HIV testing and linkage to HIV care and treatment among refugee and displaced youth [58-60]. Routine HIV testing is key to these cascades [61,62]. We address the World Health Organization (WHO) 2016-2021 HIV Global Health Sector Strategies to address youth key populations; low- and middle-income countries; and targeted HIV testing [58,59]. Community partners and knowledge users will be involved in all stages of trial design, conduct, and analysis, as well as dissemination. Should findings indicate increased effectiveness of HIV self-testing over traditional HIV testing strategies, these partnerships will facilitate the scale-up of HIV self-testing implementation for marginalized communities in Uganda.

### Ethics

The study population includes young adults (aged 16 years or above) capable of providing informed consent; Uganda’s HIV and AIDS Prevention and Control Act permits youth aged 12 years or above to independently access HIV testing and counselling without parental permission. All participants will receive information about the study before being enrolled to ensure understanding of rights for refusal/withdrawal, study processes, and expectations, and to provide written informed consent. At any time during the study data collection period, participants can withdraw from the study before completing the interview with no adverse consequences on the care or services they receive. All data will be stored on password-protected computers. To maintain confidentiality, all participants will be given a unique case ID, and no personal identifying information will be stored with the study data.

The risks associated with the Tushirikiane trial are reasonable. Physical risks exist for participants who conduct HIV testing (standard of care) and/or confirmatory testing (HIV self-testing and HIV self-testing plus mHealth). However, in all cases, HIV testing and confirmatory testing are optional. Further, the results will only be linked to participant ID. Second, an HIV diagnosis may cause stress, anxiety, and fear of stigmatization among participants. Such risks will be mitigated by the clinics conducting testing, which offer confidential pretest and posttest counselling, as well as HIV treatment. All participants will also be provided with a list of community resources regardless of HIV testing outcomes.

Any adverse event will be reported by the peer navigator to the research assistant, who will fill out an adverse event reporting form (Adverse Event Reporting Form) and adverse event narrative form if appropriate (Adverse Event Narrative Form). Adverse events can also be directly reported by study participants via a Tushirikiane hotline, which will be shared with the study participants at enrollment (Template HIV Counselor Hotline Card), can be called toll free, and will be monitored by trained HIV counselors throughout the duration of the study. Any adverse event requiring a narrative form will be reported to the principal investigators within 24 hours.

### Dissemination

Regardless of the outcomes, trial results will be published in peer-reviewed scientific journals and disseminated via many methods. The findings will be disseminated (1) to academics and researchers in HIV, sexual health, social work, and adolescent health via presentations at key scientific conferences; (2) to international collaborating organizations, with executive summaries and reports disseminated to UNAIDS, WHO, and United Nations High Commissioner for Refugees; (3) to local organizations, with reports disseminated to the Ugandan National AIDS Control Program, Ministry of Health, and our collaborators; and (4) through a research brief with highlights of the findings in all five languages.

### Data Sharing

The final data set will consist of self-reported demographic and social-ecological data from interviews with the participants and laboratory data from HIV confirmatory tests for HIV-positive individuals. Even though the final data set will be deidentified before release for sharing, it is possible to deductively disclose subjects using a combination of common characteristics. Therefore, to access our data, users need to meet our data-sharing agreement that provides for (1) the ability to secure ethics approval from both the user’s institution and the University of Toronto research ethics board; (2) a commitment to use the data solely for research purposes and to not identify any individual participant; (3) a dedication to securing the data using appropriate computer technology such as encryption; and (4) a commitment to destroying or returning the data after analyses are completed.

### Strengths and Limitations of This Study

The Tushirikiane study is unique in exploring HIV self-testing feasibility and uptake among urban refugee and displaced youth in Kampala, Uganda. Little is known about the preferred HIV testing strategies in this population.

Our three-arm cRCT longitudinal design will allow us to examine changes over time and assess if HIV self-testing alone or HIV self-testing alongside an mHealth component (bidirectional supportive SMS) increases HIV testing uptake and status awareness in comparison with the standard of care.

Our cluster randomization by informal settlements (“slums”) mitigates threats to internal validity and experimental contamination due to the shared social and physical environments between youth in the same or nearby informal settlement.

The primary study limitations are loss to follow-up, missing data points, and study delays due to COVID-19.

Our research will provide gender- and age-stratified analyses, as well as an understanding of the potential added benefits of SMS support strategies alongside HIV self-testing to inform differentiated HIV testing strategies among urban refugee and displaced youth, which can be adapted for diverse contexts.

## Appendix

Multimedia Appendix 1 CONSORT-EHEALTH checklist (V.1.6.1).

Multimedia Appendix 2 CIHR Peer Reviews.

## Abbreviations

cRCT: cluster randomized controlled trial
SRH: sexual and reproductive health
UNAIDS: Joint United Nations Program on HIV/AIDS
WHO: World Health Organization
YARID: Young African Refugees for Integral Development
