# Supplementary material for: Mobile Health–Supported HIV Self-Testing Strategy Among Urban Refugee and Displaced Youth in Kampala, Uganda: Protocol for a Cluster Randomized Trial (Tushirikiane, Supporting Each Other)
Source: JMIR Res Protoc. 2021 Feb 2;10(2):e26192. doi: 10.2196/26192 (PMC7886618; doi:10.2196/26192)
Supplement: Multimedia Appendix 1 [file resprot_v10i2e26192_app1.pdf]

CONSORT-EHEALTH (V 1.6.1) - Submission/Publication Form

The CONSORT-EHEALTH checklist is intended for authors of randomized trials evaluating web-based and Internet-based applications/interventions, including mobile interventions, electronic games (incl multiplayer games), social media, certain telehealth applications, and other interactive and/or networked electronic applications. Some of the items (e.g. all subitems under item 5 - description of the intervention) may also be applicable for other study designs.

The goal of the CONSORT EHEALTH checklist and guideline is to be
a) a guide for reporting for authors of RCTs,
b) to form a basis for appraisal of an ehealth trial (in terms of validity)

CONSORT-EHEALTH items/subitems are MANDATORY reporting items for studies published in the Journal of Medical Internet Research and other journals / scientific societies endorsing the checklist.

Items numbered 1., 2., 3., 4a., 4b etc are original CONSORT or CONSORT-NPT (non-pharmacologic treatment) items.
Items with Roman numerals (i., ii, iii, iv etc.) are CONSORT-EHEALTH extensions/clarifications.

As the CONSORT-EHEALTH checklist is still considered in a formative stage, we would ask that you also RATE ON A SCALE OF 1-5 how important/useful you feel each item is FOR THE PURPOSE OF THE CHECKLIST and reporting guideline (optional).

Mandatory reporting items are marked with a red *.
In the textboxes, either copy & paste the relevant sections from your manuscript into this form - please include any quotes from your manuscript in QUOTATION MARKS,
or answer directly by providing additional information not in the manuscript, or elaborating on why the item was not relevant for this study.

YOUR ANSWERS WILL BE PUBLISHED AS A SUPPLEMENTARY FILE TO YOUR PUBLICATION IN JMIR AND ARE CONSIDERED PART OF YOUR PUBLICATION (IF ACCEPTED).
Please fill in these questions diligently. Information will not be copyedited, so please use proper spelling and grammar, use correct capitalization, and avoid abbreviations.

DO NOT FORGET TO SAVE AS PDF _AND_ CLICK THE SUBMIT BUTTON SO YOUR ANSWERS

ARE IN OUR DATABASE !!!

Citation Suggestion (if you append the pdf as Appendix we suggest to cite this paper in the caption):
Eysenbach G, CONSORT-EHEALTH Group
CONSORT-EHEALTH: Improving and Standardizing Evaluation Reports of Web-based and Mobile Health Interventions
J Med Internet Res 2011;13(4):e126
URL: http://www.jmir.org/2011/4/e126/
doi: 10.2196/jmir.1923
PMID: 22209829

*Required

Your name *

First Last

Carmen Logie

Primary Affiliation (short), City, Country *

University of Toronto, Toronto, Canada

University of Toronto, Toronto, Canada

Your e-mail address *

abc@gmail.com

carmen.logie@utoronto.ca

Title of your manuscript *

Provide the (draft) title of your manuscript.

Mobile Health–Supported HIV Self-Testing Strategy Among Urban Refugee and Displaced Youth in Kampala, Uganda: Protocol for a Cluster Randomized Trial (Tushirikiane, Supporting Each Other)

Name of your App/Software/Intervention *

If there is a short and a long/alternate name, write the short name first and add the long name in brackets.

mobile phone app (WelTel)

Evaluated Version (if any)

e.g. "V1", "Release 2017-03-01", "Version 2.0.27913"

n/a

Language(s) *

What language is the intervention/app in? If multiple languages are available, separate by comma (e.g. "English, French")

English

URL of your Intervention Website or App

e.g. a direct link to the mobile app on app in appstore (itunes, Google Play), or URL of the website. If the intervention is a DVD or hardware, you can also link to an Amazon page.

https://www.weltelhealth.com/Home

URL of an image/screenshot (optional)

Your answer

Accessibility *

Can an enduser access the intervention presently?

- ○ access is free and open
- ◉ access only for special usergroups, not open
- ○ access is open to everyone, but requires payment/subscription/in-app purchases
- ○ app/intervention no longer accessible
- ○ Other:

Primary Medical Indication/Disease/Condition *

e.g. "Stress", "Diabetes", or define the target group in brackets after the condition, e.g. "Autism (Parents of children with)", "Alzheimers (Informal Caregivers of)"

HIV infection (young refugees)

Primary Outcomes measured in trial *

comma-separated list of primary outcomes reported in the trial

HIV testing frequency, HIV status knowledge, l

Secondary/other outcomes

Are there any other outcomes the intervention is expected to affect?

depression, condom use efficacy, consistent condom use, sexual relationship power, HIV stigma, and adolescent sexual and reproductive health stigma

Recommended "Dose" *

What do the instructions for users say on how often the app should be used?

- ○ Approximately Daily
- ◉ Approximately Weekly
- ○ Approximately Monthly
- ○ Approximately Yearly
- ○ "as needed"
- ○ Other:

Approx. Percentage of Users (starters) still using the app as recommended after 3 months *

- (x) unknown / not evaluated
- ( ) 0-10%
- ( ) 11-20%
- ( ) 21-30%
- ( ) 31-40%
- ( ) 41-50%
- ( ) 51-60%
- ( ) 61-70%
- ( ) 71%-80%
- ( ) 81-90%
- ( ) 91-100%
- ( ) Other:

Overall, was the app/intervention effective? *

- ○ yes: all primary outcomes were significantly better in intervention group vs control
- ○ partly: SOME primary outcomes were significantly better in intervention group vs control
- ○ no statistically significant difference between control and intervention
- ○ potentially harmful: control was significantly better than intervention in one or more outcomes
- ○ inconclusive: more research is needed
- ◉ Other: this is a protocol paper, we don't have this information yet

Article Preparation Status/Stage *

At which stage in your article preparation are you currently (at the time you fill in this form)

- ○ not submitted yet - in early draft status
- ○ not submitted yet - in late draft status, just before submission
- ○ submitted to a journal but not reviewed yet
- ○ submitted to a journal and after receiving initial reviewer comments
- ◉ submitted to a journal and accepted, but not published yet
- ○ published
- ○ Other:

Journal *

If you already know where you will submit this paper (or if it is already submitted), please provide the journal name (if it is not JMIR, provide the journal name under "other")

- ○ not submitted yet / unclear where I will submit this
- ○ Journal of Medical Internet Research (JMIR)
- ○ JMIR mHealth and UHealth
- ○ JMIR Serious Games
- ○ JMIR Mental Health
- ○ JMIR Public Health
- ○ JMIR Formative Research
- ◉ Other JMIR sister journal
- ○ Other:

Is this a full powered effectiveness trial or a pilot/feasibility trial? *

- ○ Pilot/feasibility
- ◉ Fully powered

Manuscript tracking number *

If this is a JMIR submission, please provide the manuscript tracking number under "other" (The ms tracking number can be found in the submission acknowledgement email, or when you login as author in JMIR. If the paper is already published in JMIR, then the ms tracking number is the four-digit number at the end of the DOI, to be found at the bottom of each published article in JMIR)

- ○ no ms number (yet) / not (yet) submitted to / published in JMIR
- ◉ Other: JRP ms#26192

TITLE AND ABSTRACT

1a) TITLE: Identification as a randomized trial in the title

1a) Does your paper address CONSORT item 1a? *

I.e does the title contain the phrase "Randomized Controlled Trial"? (if not, explain the reason under "other")

- ◉ yes
- ○ Other:

1a-i) Identify the mode of delivery in the title

Identify the mode of delivery. Preferably use “web-based” and/or “mobile” and/or “electronic game” in the title. Avoid ambiguous terms like “online”, “virtual”, “interactive”. Use “Internet-based” only if Intervention includes non-web-based Internet components (e.g. email), use “computer-based” or “electronic” only if offline products are used. Use “virtual” only in the context of “virtual reality” (3-D worlds). Use “online” only in the context of “online support groups”. Complement or substitute product names with broader terms for the class of products (such as “mobile” or “smart phone” instead of “iphone”), especially if the application runs on different platforms.

	1	2	3	4	5	
subitem not at all important	○	○	○	◉	○	essential

Clear selection

Does your paper address subitem 1a-i? *

Copy and paste relevant sections from manuscript title (include quotes in quotation marks "like this" to indicate direct quotes from your manuscript), or elaborate on this item by providing additional information not in the ms, or briefly explain why the item is not applicable/relevant for your study

yes: Mobile Health–Supported HIV Self-Testing Strategy

1a-ii) Non-web-based components or important co-interventions in title

Mention non-web-based components or important co-interventions in title, if any (e.g., “with telephone support”).

	1	2	3	4	5	
subitem not at all important	○	○	○	◉	○	essential

Clear selection

Does your paper address subitem 1a-ii?

Copy and paste relevant sections from manuscript title (include quotes in quotation marks "like this" to indicate direct quotes from your manuscript), or elaborate on this item by providing additional information not in the ms, or briefly explain why the item is not applicable/relevant for your study

yes: HIV Self-Testing Strategy

1a-iii) Primary condition or target group in the title

Mention primary condition or target group in the title, if any (e.g., "for children with Type I Diabetes") Example: A Web-based and Mobile Intervention with Telephone Support for Children with Type I Diabetes: Randomized Controlled Trial

	1	2	3	4	5	
subitem not at all important	○	○	○	●	○	essential

Clear selection

Does your paper address subitem 1a-iii? *

Copy and paste relevant sections from manuscript title (include quotes in quotation marks "like this" to indicate direct quotes from your manuscript), or elaborate on this item by providing additional information not in the ms, or briefly explain why the item is not applicable/relevant for your study

yes: Urban Refugee and Displaced Youth in Kampala,

1b) ABSTRACT: Structured summary of trial design, methods, results, and conclusions

NPT extension: Description of experimental treatment, comparator, care providers, centers, and blinding status.

1b-i) Key features/functionalities/components of the intervention and comparator in the METHODS section of the ABSTRACT

Mention key features/functionalities/components of the intervention and comparator in the abstract. If possible, also mention theories and principles used for designing the site. Keep in mind the needs of systematic reviewers and indexers by including important synonyms. (Note: Only report in the abstract what the main paper is reporting. If this information is missing from the main body of text, consider adding it)

	1	2	3	4	5	
subitem not at all important	○	○	◉	○	○	essential

Clear selection

Does your paper address subitem 1b-i? *

Copy and paste relevant sections from the manuscript abstract (include quotes in quotation marks "like this" to indicate direct quotes from your manuscript), or elaborate on this item by providing additional information not in the ms, or briefly explain why the item is not applicable/relevant for your study

yes but in objective: This study aims to evaluate the feasibility and effectiveness of two HIV self-testing implementation strategies (HIV
self-testing intervention alone and HIV self-testing combined with an mHealth intervention) in comparison with the HIV testing
standard of care in terms of HIV testing outcomes among refugee/displaced youth aged 16 to 24 years in Kampala, Uganda.

1b-ii) Level of human involvement in the METHODS section of the ABSTRACT

Clarify the level of human involvement in the abstract, e.g., use phrases like “fully automated” vs. “therapist/nurse/care provider/physician-assisted” (mention number and expertise of providers involved, if any). (Note: Only report in the abstract what the main paper is reporting. If this information is missing from the main body of text, consider adding it)

	1	2	3	4	5	
subitem not at all important	(•)	( )	( )	( )	( )	essential

Clear selection

Does your paper address subitem 1b-ii?

Copy and paste relevant sections from the manuscript abstract (include quotes in quotation marks "like this" to indicate direct quotes from your manuscript), or elaborate on this item by providing additional information not in the ms, or briefly explain why the item is not applicable/relevant for your study

No

1b-iii) Open vs. closed, web-based (self-assessment) vs. face-to-face assessments in the METHODS section of the ABSTRACT

Mention how participants were recruited (online vs. offline), e.g., from an open access website or from a clinic or a closed online user group (closed usergroup trial), and clarify if this was a purely web-based trial, or there were face-to-face components (as part of the intervention or for assessment). Clearly say if outcomes were self-assessed through questionnaires (as common in web-based trials). Note: In traditional offline trials, an open trial (open-label trial) is a type of clinical trial in which both the researchers and participants know which treatment is being administered. To avoid confusion, use “blinded” or “unblinded” to indicated the level of blinding instead of “open”, as “open” in web-based trials usually refers to “open access” (i.e. participants can self-enrol). (Note: Only report in the abstract what the main paper is reporting. If this information is missing from the main body of text, consider adding it)

	1	2	3	4	5	
subitem not at all important	(●)	( )	( )	( )	( )	essential

Clear selection

Does your paper address subitem 1b-iii?

Copy and paste relevant sections from the manuscript abstract (include quotes in quotation marks "like this" to indicate direct quotes from your manuscript), or elaborate on this item by providing additional information not in the ms, or briefly explain why the item is not applicable/relevant for your study

yes: A three-arm cluster randomized controlled trial will be implemented across five informal settlements grouped into
three sites, based on proximity, and randomization will be performed with a 1:1:1 method.

1b-iv) RESULTS section in abstract must contain use data

Report number of participants enrolled/assessed in each group, the use/uptake of the intervention (e.g., attrition/adherence metrics, use over time, number of logins etc.), in addition to primary/secondary outcomes. (Note: Only report in the abstract what the main paper is reporting. If this information is missing from the main body of text, consider adding it)

	1	2	3	4	5	
subitem not at all important	(•)	( )	( )	( )	( )	essential

Clear selection

Does your paper address subitem 1b-iv?

Copy and paste relevant sections from the manuscript abstract (include quotes in quotation marks "like this" to indicate direct quotes from your manuscript), or elaborate on this item by providing additional information not in the ms, or briefly explain why the item is not applicable/relevant for your study

No: this is a protocol paper

1b-v) CONCLUSIONS/DISCUSSION in abstract for negative trials

Conclusions/Discussions in abstract for negative trials: Discuss the primary outcome - if the trial is negative (primary outcome not changed), and the intervention was not used, discuss whether negative results are attributable to lack of uptake and discuss reasons. (Note: Only report in the abstract what the main paper is reporting. If this information is missing from the main body of text, consider adding it)

	1	2	3	4	5	
subitem not at all important	(•)	( )	( )	( )	( )	essential

Clear selection

Does your paper address subitem 1b-v?

Copy and paste relevant sections from the manuscript abstract (include quotes in quotation marks "like this" to indicate direct quotes from your manuscript), or elaborate on this item by providing additional information not in the ms, or briefly explain why the item is not applicable/relevant for your study

No: this is a protocol paper

INTRODUCTION

2a) In INTRODUCTION: Scientific background and explanation of rationale

2a-i) Problem and the type of system/solution

Describe the problem and the type of system/solution that is object of the study: intended as stand-alone intervention vs. incorporated in broader health care program? Intended for a particular patient population? Goals of the intervention, e.g., being more cost-effective to other interventions, replace or complement other solutions? (Note: Details about the intervention are provided in "Methods" under 5)

	1	2	3	4	5	
subitem not at all important	○	○	●	○	○	essential

Clear selection

Does your paper address subitem 2a-i? *

Copy and paste relevant sections from the manuscript (include quotes in quotation marks "like this" to indicate direct quotes from your manuscript), or elaborate on this item by providing additional information not in the ms, or briefly explain why the item is not applicable/relevant for your study

yes: Mobile health (mHealth) strategies (digital media on mobile devices) have been efficacious in improving antiretroviral therapy adherence and HIV and sexually transmitted infection testing, and are relevant for the way youth learn and socialize [24-26]. In particular, two-way SMS-based mHealth interventions that are interactive and supportive have been found to be more efficacious in increasing adherence than one-way messages/reminders [27,28]. However, few studies have integrated mHealth into HIV self-testing delivery with adolescents or focused on optimal HIV self-testing delivery strategies with displaced/refugee youth.

2a-ii) Scientific background, rationale: What is known about the (type of) system

Scientific background, rationale: What is known about the (type of) system that is the object of the study (be sure to discuss the use of similar systems for other conditions/diagnoses, if appropiate), motivation for the study, i.e. what are the reasons for and what is the context for this specific study, from which stakeholder viewpoint is the study performed, potential impact of findings [2]. Briefly justify the choice of the comparator.

	1	2	3	4	5	
subitem not at all important	○	○	●	○	○	essential

Clear selection

Does your paper address subitem 2a-ii? *

Copy and paste relevant sections from the manuscript (include quotes in quotation marks "like this" to indicate direct quotes from your manuscript), or elaborate on this item by providing additional information not in the ms, or briefly explain why the item is not applicable/relevant for your study

yes: Mobile health (mHealth) strategies (digital media on mobile devices) have been efficacious in improving antiretroviral therapy adherence and HIV and sexually transmitted infection testing, and are relevant for the way youth learn and socialize [24-26]. In particular, two-way SMS-based mHealth interventions that are interactive and supportive have been found to be more efficacious in increasing adherence than one-way messages/reminders [27,28]. However, few studies have integrated mHealth into HIV self-testing delivery with adolescents or focused on optimal HIV self-testing delivery strategies with displaced/refugee youth.

2b) In INTRODUCTION: Specific objectives or hypotheses

Does your paper address CONSORT subitem 2b? *

Copy and paste relevant sections from the manuscript (include quotes in quotation marks "like this" to indicate direct quotes from your manuscript), or elaborate on this item by providing additional information not in the ms, or briefly explain why the item is not applicable/relevant for your study

yes: The specific objectives of this study are
to determine the effectiveness of the interventions on the following criteria: (1) increased frequency of HIV testing; (2) increased knowledge of HIV status; (3) increased linkage to confirmatory testing (for those with an HIV positive self-test); and (4) increased linkage to HIV care (for persons testing positive for HIV in HIV self-testing and confirmatory testing).
Secondary outcomes include (1) depression, (2) condom use self-efficacy, (3) consistent condom use, (4) sexual relationship power, (5) HIV stigma, and (6) adolescent sexual and reproductive health (SRH) stigma.

METHODS

3a) Description of trial design (such as parallel, factorial) including allocation ratio

Does your paper address CONSORT subitem 3a? *

Copy and paste relevant sections from the manuscript (include quotes in quotation marks "like this" to indicate direct quotes from your manuscript), or elaborate on this item by providing additional information not in the ms, or briefly explain why the item is not applicable/relevant for your study

yes: To evaluate the intervention effectiveness, we will conduct a cluster randomized controlled trial (cRCT), where informal settlements are randomized. The clusters include five informal settlements grouped into three sites that are randomized in a 1:1:1 method to one of three study arms. Although outcome data will be collected at the level of the individual, we selected cluster randomization over individual randomization because the intervention is implemented at the settlement level. A cluster randomized design addresses threats of internal validity. It reduces the possibility of experimental contamination due to the shared social and physical environments between youth in the same or nearby informal settlements [35].

3b) Important changes to methods after trial commencement (such as eligibility criteria), with reasons

Does your paper address CONSORT subitem 3b? *

Copy and paste relevant sections from the manuscript (include quotes in quotation marks "like this" to indicate direct quotes from your manuscript), or elaborate on this item by providing additional information not in the ms, or briefly explain why the item is not applicable/relevant for your study

No: it is a protocol

3b-i) Bug fixes, Downtimes, Content Changes

Bug fixes, Downtimes, Content Changes: ehealth systems are often dynamic systems. A description of changes to methods therefore also includes important changes made on the intervention or comparator during the trial (e.g., major bug fixes or changes in the functionality or content) (5-iii) and other "unexpected events" that may have influenced study design such as staff changes, system failures/downtimes, etc. [2].

	1	2	3	4	5	
subitem not at all important	◉	○	○	○	○	essential

Clear selection

Does your paper address subitem 3b-i?

Copy and paste relevant sections from the manuscript (include quotes in quotation marks "like this" to indicate direct quotes from your manuscript), or elaborate on this item by providing additional information not in the ms, or briefly explain why the item is not applicable/relevant for your study

No, it is not relevant as this is a protocol paper

4a) Eligibility criteria for participants

Does your paper address CONSORT subitem 4a? *

Copy and paste relevant sections from the manuscript (include quotes in quotation marks "like this" to indicate direct quotes from your manuscript), or elaborate on this item by providing additional information not in the ms, or briefly explain why the item is not applicable/relevant for your study

yes: Individuals are eligible for inclusion if they meet
the following criteria: (1) currently live in one of the following
five informal settlements in Kampala: Kabalagala, Kansanga,
Katwe, Nsambya, and Rubaga; (2) identify as a
refugee/displaced person or have refugee/displaced parents; (3)
are aged 16 to 24 years; (4) speak English, Swahili, Luganda,
French, Kinyarwanda, or Kirundi; and (5) own a mobile phone.

4a-i) Computer / Internet literacy

Computer / Internet literacy is often an implicit “de facto” eligibility criterion - this should be explicitly clarified.

	1	2	3	4	5	
subitem not at all important	(•)	( )	( )	( )	( )	essential

Clear selection

Does your paper address subitem 4a-i?

Copy and paste relevant sections from the manuscript (include quotes in quotation marks "like this" to indicate direct quotes from your manuscript), or elaborate on this item by providing additional information not in the ms, or briefly explain why the item is not applicable/relevant for your study

no, it is not needed. but what is needed is mobile phone ownership that we include as stated above: Individuals are eligible for inclusion if they meet the following criteria: (1) currently live in one of the following five informal settlements in Kampala: Kabalagala, Kansanga, Katwe, Nsambya, and Rubaga; (2) identify as a refugee/displaced person or have refugee/displaced parents; (3) are aged 16 to 24 years; (4) speak English, Swahili, Luganda, French, Kinyarwanda, or Kirundi; and (5) own a mobile phone.

4a-ii) Open vs. closed, web-based vs. face-to-face assessments:

Open vs. closed, web-based vs. face-to-face assessments: Mention how participants were recruited (online vs. offline), e.g., from an open access website or from a clinic, and clarify if this was a purely web-based trial, or there were face-to-face components (as part of the intervention or for assessment), i.e., to what degree got the study team to know the participant. In online-only trials, clarify if participants were quasi-anonymous and whether having multiple identities was possible or whether technical or logistical measures (e.g., cookies, email confirmation, phone calls) were used to detect/prevent these.

	1	2	3	4	5	
subitem not at all important	○	◉	○	○	○	essential

Clear selection

Does your paper address subitem 4a-ii? *

Copy and paste relevant sections from the manuscript (include quotes in quotation marks "like this" to indicate direct quotes from your manuscript), or elaborate on this item by providing additional information not in the ms, or briefly explain why the item is not applicable/relevant for your study

yes: Participants will be recruited within each settlement using purposive methods, including word-of-mouth and venue-based sampling at refugee agencies and community events. Peer navigators will conduct peer-driven recruitment at each site, sharing youth-designed flyers for potential participants to contact (via email and mobile number) peer navigators to join the study. Community collaborators and peer navigators will facilitate participant retention. Specifically, peer navigators will use multiple study reminder strategies (eg, social media and texts) to maintain engagement. We will utilize existing outreach and services by local refugee agencies and community partners.

4a-iii) Information giving during recruitment

Information given during recruitment. Specify how participants were briefed for recruitment and in the informed consent procedures (e.g., publish the informed consent documentation as appendix, see also item X26), as this information may have an effect on user self-selection, user expectation and may also bias results.

	1	2	3	4	5	
subitem not at all important	○	◉	○	○	○	essential

Clear selection

Does your paper address subitem 4a-iii?

Copy and paste relevant sections from the manuscript (include quotes in quotation marks "like this" to indicate direct quotes from your manuscript), or elaborate on this item by providing additional information not in the ms, or briefly explain why the item is not applicable/relevant for your study

yes: Participants will be recruited within each settlement using purposive methods, including word-of-mouth and venue-based sampling at refugee agencies and community events. Peer navigators will conduct peer-driven recruitment at each site, sharing youth-designed flyers for potential participants to contact (via email and mobile number) peer navigators to join the study. Community collaborators and peer navigators will facilitate participant retention. Specifically, peer navigators will use multiple study reminder strategies (eg, social media and texts) to maintain engagement. We will utilize existing outreach and services by local refugee agencies and community partners. Additionally, this project engages peer navigators who identify as refugees or displaced persons (aged 18-24 years) to help with participant recruitment and to provide feedback on the study design and survey. Twelve peer navigators (six young women and six young men) recruited for this study have been identified by community-based collaborators, have experience working in the various study communities as health or peer educators, and are deeply respected and connected in their communities.

4b) Settings and locations where the data were collected

Does your paper address CONSORT subitem 4b? *

Copy and paste relevant sections from the manuscript (include quotes in quotation marks "like this" to indicate direct quotes from your manuscript), or elaborate on this item by providing additional information not in the ms, or briefly explain why the item is not applicable/relevant for your study

The five informal settlements selected for this study are grouped into three sites based on their proximity to one another (Kabalagala/Kansanga, Katwe/Nsambya, and Rubaga) and have been purposively chosen because these communities host many displaced/refugee persons in Kampala [16,18,36,37].

4b-i) Report if outcomes were (self-)assessed through online questionnaires

Clearly report if outcomes were (self-)assessed through online questionnaires (as common in web-based trials) or otherwise.

	1	2	3	4	5	
subitem not at all important	(selected)	○	○	○	○	essential

Clear selection

Does your paper address subitem 4b-i? *

Copy and paste relevant sections from the manuscript (include quotes in quotation marks "like this" to indicate direct quotes from your manuscript), or elaborate on this item by providing additional information not in the ms, or briefly explain why the item is not applicable/relevant for your study

yes: Outcome data will be collected at three time points as identified using a tablet-based survey application (QuickTapSurvey, Formstack for Time 1; SurveyCTO, Dobility for Time 2 and Time 3). Baseline data to characterize the study population include demographics and sexual history, which will be collected using these tools. Data will be automatically uploaded onto a secure password-protected server using an SSL certificate and will remain encrypted when not in use.

4b-ii) Report how institutional affiliations are displayed

Report how institutional affiliations are displayed to potential participants [on ehealth media], as affiliations with prestigious hospitals or universities may affect volunteer rates, use, and reactions with regards to an intervention.(Not a required item – describe only if this may bias results)

	1	2	3	4	5	
subitem not at all important	(●)	( )	( )	( )	( )	essential

Clear selection

Does your paper address subitem 4b-ii?

Copy and paste relevant sections from the manuscript (include quotes in quotation marks "like this" to indicate direct quotes from your manuscript), or elaborate on this item by providing additional information not in the ms, or briefly explain why the item is not applicable/relevant for your study

no, not applicable

5) The interventions for each group with sufficient details to allow replication, including how and when they were actually administered

5-i) Mention names, credential, affiliations of the developers, sponsors, and owners

Mention names, credential, affiliations of the developers, sponsors, and owners [6] (if authors/evaluators are owners or developer of the software, this needs to be declared in a "Conflict of interest" section or mentioned elsewhere in the manuscript).

	1	2	3	4	5	
subitem not at all important	○	◉	○	○	○	essential

Clear selection

Does your paper address subitem 5-i?

Copy and paste relevant sections from the manuscript (include quotes in quotation marks "like this" to indicate direct quotes from your manuscript), or elaborate on this item by providing additional information not in the ms, or briefly explain why the item is not applicable/relevant for your study

yes it is included in the conflict of interest statement.

5-ii) Describe the history/development process

Describe the history/development process of the application and previous formative evaluations (e.g., focus groups, usability testing), as these will have an impact on adoption/use rates and help with interpreting results.

	1	2	3	4	5	
subitem not at all important	○	◉	○	○	○	essential

Clear selection

Does your paper address subitem 5-ii?

Copy and paste relevant sections from the manuscript (include quotes in quotation marks "like this" to indicate direct quotes from your manuscript), or elaborate on this item by providing additional information not in the ms, or briefly explain why the item is not applicable/relevant for your study

yes: Participants in this arm will be enrolled into the HIV self-testing group (as above) and on a web-based SMS platform hosted by WelTel [40,41]. WelTel is a nonprofit agency developing the mHealth intervention, in which participants receive weekly supportive bidirectional text messages asking how they are doing [42]. Participants are requested to reply “fine” or “not fine,” and those responding “not fine” will be contacted with support by a peer navigator. Participants in this arm will discuss the weekly WelTel messages with peer navigators and respond to the “not fine” messages within 2 days. If they do not reply to the initial SMS message within the specified timeframe, a peer navigator will follow up with them during that week. The WelTel system will manage the SMS intervention on a structured mobile phone platform (all SMS interactions are logged). WelTel’s two-way texting mHealth intervention may prompt participants to engage in HIV self-testing and/or to engage peer navigator support in decision making regarding HIV self-testing practices.

5-iii) Revisions and updating

Revisions and updating. Clearly mention the date and/or version number of the application/intervention (and comparator, if applicable) evaluated, or describe whether the intervention underwent major changes during the evaluation process, or whether the development and/or content was "frozen" during the trial. Describe dynamic components such as news feeds or changing content which may have an impact on the replicability of the intervention (for unexpected events see item 3b).

	1	2	3	4	5	
subitem not at all important	(●)	( )	( )	( )	( )	essential

Clear selection

Does your paper address subitem 5-iii?

Copy and paste relevant sections from the manuscript (include quotes in quotation marks "like this" to indicate direct quotes from your manuscript), or elaborate on this item by providing additional information not in the ms, or briefly explain why the item is not applicable/relevant for your study

no: this is a protocol paper, not applicable

5-iv) Quality assurance methods

Provide information on quality assurance methods to ensure accuracy and quality of information provided [1], if applicable.

	1	2	3	4	5	
subitem not at all important	( )	(●)	( )	( )	( )	essential

Clear selection

Does your paper address subitem 5-iv?

Copy and paste relevant sections from the manuscript (include quotes in quotation marks "like this" to indicate direct quotes from your manuscript), or elaborate on this item by providing additional information not in the ms, or briefly explain why the item is not applicable/relevant for your study

yes: Regardless of the outcomes, trial results will be published in peer-reviewed scientific journals and disseminated via many
methods. The findings will be disseminated (1) to academics
and researchers in HIV, sexual health, social work, and
adolescent health via presentations at key scientific conferences;
(2) to international collaborating organizations, with executive
summaries and reports disseminated to UNAIDS, WHO, and
United Nations High Commissioner for Refugees; (3) to local
organizations, with reports disseminated to the Ugandan
National AIDS Control Program, Ministry of Health, and our
collaborators; and (4) through a research brief with highlights
of the findings in all five languages.

5-v) Ensure replicability by publishing the source code, and/or providing screenshots/screen-capture video, and/or providing flowcharts of the algorithms used

Ensure replicability by publishing the source code, and/or providing screenshots/screen-capture video, and/or providing flowcharts of the algorithms used. Replicability (i.e., other researchers should in principle be able to replicate the study) is a hallmark of scientific reporting.

	1	2	3	4	5	
subitem not at all important	◉	○	○	○	○	essential

Clear selection

Does your paper address subitem 5-v?

Copy and paste relevant sections from the manuscript (include quotes in quotation marks "like this" to indicate direct quotes from your manuscript), or elaborate on this item by providing additional information not in the ms, or briefly explain why the item is not applicable/relevant for your study

no: not applicable, this is a protocol

5-vi) Digital preservation

Digital preservation: Provide the URL of the application, but as the intervention is likely to change or disappear over the course of the years; also make sure the intervention is archived (Internet Archive, webcitation.org, and/or publishing the source code or screenshots/videos alongside the article). As pages behind login screens cannot be archived, consider creating demo pages which are accessible without login.

	1	2	3	4	5	
subitem not at all important	◉	○	○	○	○	essential

Clear selection

Does your paper address subitem 5-vi?

Copy and paste relevant sections from the manuscript (include quotes in quotation marks "like this" to indicate direct quotes from your manuscript), or elaborate on this item by providing additional information not in the ms, or briefly explain why the item is not applicable/relevant for your study

not applicable, this is a mobile phone application operated via WelTel, not openly available.

5-vii) Access

Access: Describe how participants accessed the application, in what setting/context, if they had to pay (or were paid) or not, whether they had to be a member of specific group. If known, describe how participants obtained “access to the platform and Internet” [1]. To ensure access for editors/reviewers/readers, consider to provide a “backdoor” login account or demo mode for reviewers/readers to explore the application (also important for archiving purposes, see vi).

	1	2	3	4	5	
subitem not at all important	(●)	( )	( )	( )	( )	essential

Clear selection

Does your paper address subitem 5-vii? *

Copy and paste relevant sections from the manuscript (include quotes in quotation marks "like this" to indicate direct quotes from your manuscript), or elaborate on this item by providing additional information not in the ms, or briefly explain why the item is not applicable/relevant for your study

yes: Participants in this arm will be enrolled into the HIV self-testing group (as above) and on a web-based SMS platform hosted by WelTel [40,41]. WelTel is a nonprofit agency developing the mHealth intervention, in which participants receive weekly supportive bidirectional text messages asking how they are doing [42].

5-viii) Mode of delivery, features/functionalities/components of the intervention and comparator, and the theoretical framework

Describe mode of delivery, features/functionalities/components of the intervention and comparator, and the theoretical framework [6] used to design them (instructional strategy [1], behaviour change techniques, persuasive features, etc., see e.g., [7, 8] for terminology). This includes an in-depth description of the content (including where it is coming from and who developed it) [1],” whether [and how] it is tailored to individual circumstances and allows users to track their progress and receive feedback” [6]. This also includes a description of communication delivery channels and – if computer-mediated communication is a component – whether communication was synchronous or asynchronous [6]. It also includes information on presentation strategies [1], including page design principles, average amount of text on pages, presence of hyperlinks to other resources, etc. [1].

	1	2	3	4	5	
subitem not at all important	○	◉	○	○	○	essential

Clear selection

Does your paper address subitem 5-viii? *

Copy and paste relevant sections from the manuscript (include quotes in quotation marks "like this" to indicate direct quotes from your manuscript), or elaborate on this item by providing additional information not in the ms, or briefly explain why the item is not applicable/relevant for your study

yes: Participants are requested to reply “fine” or “not fine,” and those responding “not fine” will be contacted with support by a peer navigator. Participants in this arm will discuss the weekly WelTel messages with peer navigators and respond to the “not fine” messages within 2 days. If they do not reply to the initial SMS message within the specified timeframe, a peer navigator will follow up with them during that week. The WelTel system will manage the SMS intervention on a structured mobile phone platform (all SMS interactions are logged). WelTel’s two-way texting mHealth intervention may prompt participants to engage in HIV self-testing and/or to engage peer navigator support in decision making regarding HIV self-testing practices.

5-ix) Describe use parameters

Describe use parameters (e.g., intended “doses” and optimal timing for use). Clarify what instructions or recommendations were given to the user, e.g., regarding timing, frequency, heaviness of use, if any, or was the intervention used ad libitum.

	1	2	3	4	5	
subitem not at all important	○	◉	○	○	○	essential

Clear selection

Does your paper address subitem 5-ix?

Copy and paste relevant sections from the manuscript (include quotes in quotation marks "like this" to indicate direct quotes from your manuscript), or elaborate on this item by providing additional information not in the ms, or briefly explain why the item is not applicable/relevant for your study

yes: Participants are requested to reply “fine” or “not fine,” and those responding “not fine” will be contacted with support by a peer navigator. Participants in this arm will discuss the weekly WelTel messages with peer navigators and respond to the “not fine” messages within 2 days. If they do not reply to the initial SMS message within the specified timeframe, a peer navigator will follow up with them during that week. The WelTel system will manage the SMS intervention on a structured mobile phone platform (all SMS interactions are logged). WelTel’s two-way texting mHealth intervention may prompt participants to engage in HIV self-testing and/or to engage peer navigator support in decision making regarding HIV self-testing practices.

5-x) Clarify the level of human involvement

Clarify the level of human involvement (care providers or health professionals, also technical assistance) in the e-intervention or as co-intervention (detail number and expertise of professionals involved, if any, as well as “type of assistance offered, the timing and frequency of the support, how it is initiated, and the medium by which the assistance is delivered”. It may be necessary to distinguish between the level of human involvement required for the trial, and the level of human involvement required for a routine application outside of a RCT setting (discuss under item 21 – generalizability).

	1	2	3	4	5	
subitem not at all important	◉	○	○	○	○	essential

Clear selection

Does your paper address subitem 5-x?

Copy and paste relevant sections from the manuscript (include quotes in quotation marks "like this" to indicate direct quotes from your manuscript), or elaborate on this item by providing additional information not in the ms, or briefly explain why the item is not applicable/relevant for your study

yes: Participants are requested to reply “fine” or “not fine,” and those responding “not fine” will be contacted with support by a peer navigator. Participants in this arm will discuss the weekly WelTel messages with peer navigators and respond to the “not fine” messages within 2 days. If they do not reply to the initial SMS message within the specified timeframe, a peer navigator will follow up with them during that week.

5-xi) Report any prompts/reminders used

Report any prompts/reminders used: Clarify if there were prompts (letters, emails, phone calls, SMS) to use the application, what triggered them, frequency etc. It may be necessary to distinguish between the level of prompts/reminders required for the trial, and the level of prompts/reminders for a routine application outside of a RCT setting (discuss under item 21 – generalizability).

	1	2	3	4	5	
subitem not at all important	○	◉	○	○	○	essential

Clear selection

Does your paper address subitem 5-xi? *

Copy and paste relevant sections from the manuscript (include quotes in quotation marks "like this" to indicate direct quotes from your manuscript), or elaborate on this item by providing additional information not in the ms, or briefly explain why the item is not applicable/relevant for your study

yes: Participants are requested to reply “fine” or “not fine,” and those responding “not fine” will be contacted with support by a peer navigator. Participants in this arm will discuss the weekly WelTel messages with peer navigators and respond to the “not fine” messages within 2 days. If they do not reply to the initial SMS message within the specified timeframe, a peer navigator will follow up with them during that week.

5-xii) Describe any co-interventions (incl. training/support)

Describe any co-interventions (incl. training/support): Clearly state any interventions that are provided in addition to the targeted eHealth intervention, as ehealth intervention may not be designed as stand-alone intervention. This includes training sessions and support [1]. It may be necessary to distinguish between the level of training required for the trial, and the level of training for a routine application outside of a RCT setting (discuss under item 21 – generalizability.

	1	2	3	4	5	
subitem not at all important	○	◉	○	○	○	essential

Clear selection

Does your paper address subitem 5-xii? *

Copy and paste relevant sections from the manuscript (include quotes in quotation marks "like this" to indicate direct quotes from your manuscript), or elaborate on this item by providing additional information not in the ms, or briefly explain why the item is not applicable/relevant for your study

yes: Treatment Arm 1: HIV Self-Testing Intervention
Participants in this arm will be enrolled into the HIV self-testing intervention group and will receive HIV self-testing kits so they can perform HIV testing. At baseline enrollment, the peer navigator will provide an HIV self-testing kit (OraQuick Rapid HIV-1/2 Antibody Test, OraSure Technologies), which includes an oral swab test stick and tube solutions, a written detailed step-by-step description of how to correctly use the HIV self-testing kit, pictorial and written guides for the HIV self-testing kit, condoms and lubricant, information booklets on HIV and testing, and referral cards with the addresses and phone numbers of local clinics for confirmatory testing. The cards will also have the peer navigator's phone number to allow participants to contact the peer navigator if they need additional information on how to use the kit or need support to go for confirmatory tests at local clinics. Instructions for the kit are in French, Luganda, Swahili, Kirundi, Kinyarwanda, and English and have been pilot tested for clarity and comprehensiveness with peer navigators. A 24-hour contact number will be provided to participants to text if/when they have questions. At follow-up visits, peer navigators will check in with participants about the HIV self-testing kit, distribute another HIV self-testing kit and condoms/lubricant, and screen for adverse events (eg, negative HIV self-testing–related experiences).

6a) Completely defined pre-specified primary and secondary outcome measures, including how and when they were assessed

Does your paper address CONSORT subitem 6a? *

Copy and paste relevant sections from the manuscript (include quotes in quotation marks "like this" to indicate direct quotes from your manuscript), or elaborate on this item by providing additional information not in the ms, or briefly explain why the item is not applicable/relevant for your study

yes: This study aims to evaluate the feasibility and clinical effectiveness of two HIV self-testing delivery approaches (HIV self-testing intervention alone and HIV self-testing combined with a two-way supportive SMS mHealth intervention) in comparison with the standard of care in terms of HIV testing uptake among refugee and displaced youth aged 16 to 24 years in Kampala, Uganda. The specific objectives of this study are to determine the effectiveness of the interventions on the following criteria: (1) increased frequency of HIV testing; (2) increased knowledge of HIV status; (3) increased linkage to confirmatory testing (for those with an HIV positive self-test); and (4) increased linkage to HIV care (for persons testing positive for HIV in HIV self-testing and confirmatory testing).
Secondary outcomes include (1) depression, (2) condom use self-efficacy, (3) consistent condom use, (4) sexual relationship power, (5) HIV stigma, and (6) adolescent sexual and reproductive health (SRH) stigma.
The primary outcomes measured in this trial are as follows:
1. Changes in HIV testing frequency: This is measured as participants' self-reported last HIV test. To capture changes, this measure is assessed at all three study time points (baseline [Time 1], 8 months [Time 2], and 12 months [Time 3]).
2. Changes in knowledge of HIV status: To address social desirability bias challenges regarding self-reported HIV serostatus, multiple steps are employed. First, at each timepoint (Time 1, Time 2, and Time 3), participants are asked their current HIV status. At Time 3, participants are offered a completely voluntary rapid HIV test. Knowledge of HIV status will be assessed as correct for persons who

agree to take the rapid test and correctly report their HIV status (prior to receiving the result).
3. Changes in linkage to confirmatory HIV testing: Participants in trial arms 1 and 2 (HIV self-testing and mHealth HIV self-testing intervention) are asked at Time 2 and Time 3 if they used their HIV self-testing kit. For those who respond affirmatively, they will be asked the result, and those who report a positive result will be asked if and where they received confirmatory testing.
4. Changes in linkage to HIV care: Participants who seroconvert during the study are asked the frequency of accessing HIV care service since diagnosis. This will be assessed at Time 2 and Time 3.
5. HIV self-testing kit use: To understand the frequency of HIV self-testing kit use and to reduce social desirability bias regarding HIV self-testing kit use, participants in trial arms 1 and 2 will be followed up 1 month after Time 3 to request for purchasing unused kits back. Participants will not be informed of this as an option prior to this time.
Secondary Outcomes
Secondary outcomes include changes in depression assessed using the Patient Health Questionnaire-9 items (PHQ-9) [43]; condom use self-efficacy measured with the Condom Use Efficacy Scale [44,45]; consistent condom use frequency assessed by asking participants if they used condoms every time (consistently) in the past 3 months; sexual relationship power assessed using the relationship control subscale from the Sexual and Reproductive Power Scale [46]; perceived HIV-related stigma with the perceived HIV-related stigma subscale of Steward et al [47]; and adolescent SRH stigma assessed with the Ugandan Adolescent SRH Stigma Scale [48] adapted from the Adolescent SRH scale by Hall et al [49].

6a-i) Online questionnaires: describe if they were validated for online use and apply CHERRIES items to describe how the questionnaires were designed/deployed

If outcomes were obtained through online questionnaires, describe if they were validated for online use and apply CHERRIES items to describe how the questionnaires were designed/deployed [9].

	1	2	3	4	5	
subitem not at all important	(●)	( )	( )	( )	( )	essential

Clear selection

Does your paper address subitem 6a-i?

Copy and paste relevant sections from manuscript text

no: not applicable, they are interviewer administered and not online questionnaires

6a-ii) Describe whether and how “use” (including intensity of use/dosage) was defined/measured/monitored

Describe whether and how “use” (including intensity of use/dosage) was defined/measured/monitored (logins, logfile analysis, etc.). Use/adoption metrics are important process outcomes that should be reported in any ehealth trial.

	1	2	3	4	5	
subitem not at all important	( )	(●)	( )	( )	( )	essential

Clear selection

Does your paper address subitem 6a-ii?

Copy and paste relevant sections from manuscript text

yes: The WelTel system will manage the SMS intervention on a structured mobile phone platform (all SMS interactions are logged).

6a-iii) Describe whether, how, and when qualitative feedback from participants was obtained

Describe whether, how, and when qualitative feedback from participants was obtained (e.g., through emails, feedback forms, interviews, focus groups).

	1	2	3	4	5	
subitem not at all important	○	◉	○	○	○	essential

Clear selection

Does your paper address subitem 6a-iii?

Copy and paste relevant sections from manuscript text

yes: Peer navigators, who themselves are urban refugee youth
living in Kampala's informal settlements, provided feedback
for the study design and outcomes; conducted recruitment and active engagement
with study participants for retention;
supported study implementation by facilitating linkages between
participants and data collectors; pilot tested the survey to assess
the time required to participate in the research; and will engage
in multiple dissemination strategies for community members
(eg, providing input for infographic design and sharing
community reports with community stakeholders including the
Ministry of Health).

6b) Any changes to trial outcomes after the trial commenced, with reasons

Does your paper address CONSORT subitem 6b? *

Copy and paste relevant sections from the manuscript (include quotes in quotation marks "like this" to indicate direct quotes from your manuscript), or elaborate on this item by providing additional information not in the ms, or briefly explain why the item is not applicable/relevant for your study

yes: The Tushirikiane trial launched in February 2020, recruiting a total of 452
participants. Data collection was paused for 8 months due to COVID-19.

7a) How sample size was determined

NPT: When applicable, details of whether and how the clustering by care provides or centers was addressed

7a-i) Describe whether and how expected attrition was taken into account when calculating the sample size

Describe whether and how expected attrition was taken into account when calculating the sample size.

	1	2	3	4	5	
subitem not at all important	○	◉	○	○	○	essential

Clear selection

Does your paper address subitem 7a-i?

Copy and paste relevant sections from manuscript title (include quotes in quotation marks "like this" to indicate direct quotes from your manuscript), or elaborate on this item by providing additional information not in the ms, or briefly explain why the item is not applicable/relevant for your study

yes: Cluster sizes of 130 per group (n=390) are required to have 80%
power (P<.05) to detect a 25% difference (39% [5] vs 64%
tested) in HIV testing between any two groups from three
pairwise comparisons (control vs arm 1, control vs arm 2, and
arm 1 vs arm 2) for an odds ratio of 1.66. We assume an
intraclass correlation coefficient of 0.013 based on HIV research
on condom use in sub-Saharan Africa [50]. With 10% attrition,
432 participants (144 per cluster) are required. Computations were performed
using RStudio version 3.3.0 (RStudio Team),
based on formulae for multiple comparisons of proportions, and
adjusted for design effect [51].

7b) When applicable, explanation of any interim analyses and stopping guidelines

Does your paper address CONSORT subitem 7b? *

Copy and paste relevant sections from the manuscript (include quotes in quotation marks "like this" to indicate direct quotes from your manuscript), or elaborate on this item by providing additional information not in the ms, or briefly explain why the item is not applicable/relevant for your study

no: not applicable, this is a protocol paper.

8a) Method used to generate the random allocation sequence

NPT: When applicable, how care providers were allocated to each trial group

Does your paper address CONSORT subitem 8a? *

Copy and paste relevant sections from the manuscript (include quotes in quotation marks "like this" to indicate direct quotes from your manuscript), or elaborate on this item by providing additional information not in the ms, or briefly explain why the item is not applicable/relevant for your study

yes: The study has been designed as a three-arm cRCT consisting of two treatment arms and one control arm. Clusters will be randomized to one of the following three arms: (1) HIV self-testing, (2) HIV self-testing plus mHealth strategies (supportive text messages), and (3) standard of care (clinic-based HIV testing).

8b) Type of randomisation; details of any restriction (such as blocking and block size)

Does your paper address CONSORT subitem 8b? *

Copy and paste relevant sections from the manuscript (include quotes in quotation marks "like this" to indicate direct quotes from your manuscript), or elaborate on this item by providing additional information not in the ms, or briefly explain why the item is not applicable/relevant for your study

yes: To evaluate the intervention effectiveness, we will conduct a cluster randomized controlled trial (cRCT), where informal settlements are randomized. The clusters include five informal settlements grouped into three sites that are randomized in a 1:1:1 method to one of three study arms. Although outcome data will be collected at the level of the individual, we selected cluster randomization over individual randomization because the intervention is implemented at the settlement level. A cluster randomized design addresses threats of internal validity. It reduces the possibility of experimental contamination due to the shared social and physical environments between youth in the same or nearby informal settlements [35]. Data will be collected at the following three time points: baseline enrollment into the intervention, 8 months after enrollment, and 12 months after enrollment.

9) Mechanism used to implement the random allocation sequence (such as sequentially numbered containers), describing any steps taken to conceal the sequence until interventions were assigned

Does your paper address CONSORT subitem 9? *

Copy and paste relevant sections from the manuscript (include quotes in quotation marks "like this" to indicate direct quotes from your manuscript), or elaborate on this item by providing additional information not in the ms, or briefly explain why the item is not applicable/relevant for your study

No: not applicable, not blinded

10) Who generated the random allocation sequence, who enrolled participants, and who assigned participants to interventions

Does your paper address CONSORT subitem 10? *

Copy and paste relevant sections from the manuscript (include quotes in quotation marks "like this" to indicate direct quotes from your manuscript), or elaborate on this item by providing additional information not in the ms, or briefly explain why the item is not applicable/relevant for your study

yes: The study has been designed as a three-arm cRCT consisting of two treatment arms and one control arm. Clusters will be randomized to one of the following three arms: (1) HIV self-testing, (2) HIV self-testing plus mHealth strategies (supportive text messages), and (3) standard of care (clinic-based HIV testing). The trial arms and interventions are described below and summarized in Figure 1.
The Tushirikiane trial protocol has been approved by the Research Ethics Board of the University of Toronto (June 14, 2019), Mildmay Uganda Research Ethics Committee (November 11, 2019), and Uganda National Council for Science & Technology (August 3, 2020). The trial is registered at ClinicalTrials.gov (NCT04504097). The Tushirikiane trial launched on February 15, 2020, recruiting a total of 452 participants. Data collection was paused for 8 months owing to COVID-19. Data collection for wave 2 resumed on November 18, 2020, and as of December 10, 2020, a total of 295 participants have been followed up. Data collection for the third, and final, wave will be conducted between February and March 2021. The final follow-up to purchase back unused HIV self-testing kits will occur in June 2021.

11a) If done, who was blinded after assignment to interventions (for example, participants, care providers, those assessing outcomes) and how

NPT: Whether or not administering co-interventions were blinded to group assignment

11a-i) Specify who was blinded, and who wasn’t

Specify who was blinded, and who wasn’t. Usually, in web-based trials it is not possible to blind the participants [1, 3] (this should be clearly acknowledged), but it may be possible to blind outcome assessors, those doing data analysis or those administering co-interventions (if any).

	1	2	3	4	5	
subitem not at all important	(•)	( )	( )	( )	( )	essential

Clear selection

Does your paper address subitem 11a-i? *

Copy and paste relevant sections from the manuscript (include quotes in quotation marks "like this" to indicate direct quotes from your manuscript), or elaborate on this item by providing additional information not in the ms, or briefly explain why the item is not applicable/relevant for your study

not applicable, no blinding

11a-ii) Discuss e.g., whether participants knew which intervention was the “intervention of interest” and which one was the “comparator”

Informed consent procedures (4a-ii) can create biases and certain expectations - discuss e.g., whether participants knew which intervention was the “intervention of interest” and which one was the “comparator”.

	1	2	3	4	5	
subitem not at all important	(•)	( )	( )	( )	( )	essential

Clear selection

Does your paper address subitem 11a-ii?

Copy and paste relevant sections from the manuscript (include quotes in quotation marks "like this" to indicate direct quotes from your manuscript), or elaborate on this item by providing additional information not in the ms, or briefly explain why the item is not applicable/relevant for your study

No: not applicable, not blinded

11b) If relevant, description of the similarity of interventions

(this item is usually not relevant for ehealth trials as it refers to similarity of a placebo or sham intervention to a active medication/intervention)

Does your paper address CONSORT subitem 11b? *

Copy and paste relevant sections from the manuscript (include quotes in quotation marks "like this" to indicate direct quotes from your manuscript), or elaborate on this item by providing additional information not in the ms, or briefly explain why the item is not applicable/relevant for your study

not applicable

12a) Statistical methods used to compare groups for primary and secondary outcomes

NPT: When applicable, details of whether and how the clustering by care providers or centers was addressed

Does your paper address CONSORT subitem 12a? *

Copy and paste relevant sections from the manuscript (include quotes in quotation marks "like this" to indicate direct quotes from your manuscript), or elaborate on this item by providing additional information not in the ms, or briefly explain why the item is not applicable/relevant for your study

yes: Analysis and reporting will be conducted in accordance with CONSORT (Consolidated Standards of Reporting Trials) guidelines [52] (Multimedia Appendix 1). The analyst will be blinded to group allocation. A flow diagram will be used to illustrate patient flow (screening, randomization, allocation, and follow-up). Baseline data will be reported for all three arms and summarized as mean (SD) or median (first quartile, third quartile) for continuous variables and as count and number (percentage) for categorical variables. Primary analysis will involve intention-to-treat analysis (data from participants will be analyzed according to their allocation, irrespective of whether they actually received that intervention). Between-group comparisons will be performed using multilevel mixed effect logistic or linear regression models (to account for clustering), depending on which outcome is being evaluated. For these models, the intervention group will be entered as a fixed effect. The level of significance will be set at α=.05, but adjusted using the Bonferroni method for secondary outcomes [53]. The results will be expressed as odds ratios or mean differences as appropriate, accompanied by 95% CIs and P values. We will conduct adjusted analysis for the primary outcome (changes in HIV testing frequency) to investigate the role of various covariates in the relative effect. We will build mixed effect multilevel logistic regression models with the intervention group as the independent variable and HIV testing uptake in the past 3 months (yes/no) as the dependent variable. Covariates (eg, age) will be entered as a block. We will explore gender differences in primary and secondary intervention outcomes.

12a-i) Imputation techniques to deal with attrition / missing values

Imputation techniques to deal with attrition / missing values: Not all participants will use the intervention/comparator as intended and attrition is typically high in ehealth trials. Specify how participants who did not use the application or dropped out from the trial were treated in the statistical analysis (a complete case analysis is strongly discouraged, and simple imputation techniques such as LOCF may also be problematic [4]).

	1	2	3	4	5	
subitem not at all important	◉	○	○	○	○	essential

Clear selection

Does your paper address subitem 12a-i? *

Copy and paste relevant sections from the manuscript (include quotes in quotation marks "like this" to indicate direct quotes from your manuscript), or elaborate on this item by providing additional information not in the ms, or briefly explain why the item is not applicable/relevant for your study

no: not applicable

12b) Methods for additional analyses, such as subgroup analyses and adjusted analyses

Does your paper address CONSORT subitem 12b? *

Copy and paste relevant sections from the manuscript (include quotes in quotation marks "like this" to indicate direct quotes from your manuscript), or elaborate on this item by providing additional information not in the ms, or briefly explain why the item is not applicable/relevant for your study

yes: Covariates (eg, age) will be entered as a block. We will explore gender differences in primary and secondary intervention outcomes.

X26) REB/IRB Approval and Ethical Considerations [recommended as subheading under "Methods"] (not a CONSORT item)

X26-i) Comment on ethics committee approval

	1	2	3	4	5	
subitem not at all important	○	◉	○	○	○	essential

Clear selection

Does your paper address subitem X26-i?

Copy and paste relevant sections from the manuscript (include quotes in quotation marks "like this" to indicate direct quotes from your manuscript), or elaborate on this item by providing additional information not in the ms, or briefly explain why the item is not applicable/relevant for your study

yes: The Tushirikiane trial protocol has been approved by the Research Ethics Board of the University of Toronto (June 14, 2019), Mildmay Uganda Research Ethics Committee (November 11, 2019), and Uganda National Council for Science & Technology (August 3, 2020).

x26-ii) Outline informed consent procedures

Outline informed consent procedures e.g., if consent was obtained offline or online (how? Checkbox, etc.?), and what information was provided (see 4a-ii). See [6] for some items to be included in informed consent documents.

	1	2	3	4	5	
subitem not at all important	(•)	( )	( )	( )	( )	essential

Clear selection

Does your paper address subitem X26-ii?

Copy and paste relevant sections from the manuscript (include quotes in quotation marks "like this" to indicate direct quotes from your manuscript), or elaborate on this item by providing additional information not in the ms, or briefly explain why the item is not applicable/relevant for your study

yes: The study population includes young adults (aged 16 years or above) capable of providing informed consent; Uganda's HIV and AIDS Prevention and Control Act permits youth aged 12 years or above to independently access HIV testing and counselling without parental permission. All participants will receive information about the study before being enrolled to ensure understanding of rights for refusal/withdrawal, study processes, and expectations, and to provide written informed consent. At any time during the study data collection period, participants can withdraw from the study before completing the interview with no adverse consequences on the care or services they receive. All data will be stored on password-protected computers. To maintain confidentiality, all participants will be given a unique case ID, and no personal identifying information will be stored with the study data.

X26-iii) Safety and security procedures

Safety and security procedures, incl. privacy considerations, and any steps taken to reduce the likelihood or detection of harm (e.g., education and training, availability of a hotline)

	1	2	3	4	5	
subitem not at all important	○	○	○	○	○	essential

Does your paper address subitem X26-iii?

Copy and paste relevant sections from the manuscript (include quotes in quotation marks "like this" to indicate direct quotes from your manuscript), or elaborate on this item by providing additional information not in the ms, or briefly explain why the item is not applicable/relevant for your study

yes: The risks associated with the Tushirikiane trial are reasonable.
Physical risks exist for participants who conduct HIV testing
(standard of care) and/or confirmatory testing (HIV self-testing
and HIV self-testing plus mHealth). However, in all cases, HIV
testing and confirmatory testing are optional. Further, the results
will only be linked to participant ID. Second, an HIV diagnosis
may cause stress, anxiety, and fear of stigmatization among
participants. Such risks will be mitigated by the clinics
conducting testing, which offer confidential pretest and posttest
counselling, as well as HIV treatment. All participants will also be provided with a
list of community resources regardless of
HIV testing outcomes. Any adverse event will be reported by the peer navigator to the
research assistant, who will fill out an adverse event reporting
form (Adverse Event Reporting Form) and adverse event
narrative form if appropriate (Adverse Event Narrative Form).
Adverse events can also be directly reported by study
participants via a Tushirikiane hotline, which will be shared
with the study participants at enrollment (Template HIV
Counselor Hotline Card), can be called toll free, and will be
monitored by trained HIV counselors throughout the duration
of the study. Any adverse event requiring a narrative form will
be reported to the principal investigators within 24 hours.

RESULTS

13a) For each group, the numbers of participants who were randomly assigned, received intended treatment, and were analysed for the primary outcome

NPT: The number of care providers or centers performing the intervention in each group and the number of patients treated by each care provider in each center

Does your paper address CONSORT subitem 13a? *

Copy and paste relevant sections from the manuscript (include quotes in quotation marks "like this" to indicate direct quotes from your manuscript), or elaborate on this item by providing additional information not in the ms, or briefly explain why the item is not applicable/relevant for your study

no: not applicable, this is a protocol

13b) For each group, losses and exclusions after randomisation, together with reasons

Does your paper address CONSORT subitem 13b? (NOTE: Preferably, this is shown in a CONSORT flow diagram) *

Copy and paste relevant sections from the manuscript (include quotes in quotation marks "like this" to indicate direct quotes from your manuscript), or elaborate on this item by providing additional information not in the ms, or briefly explain why the item is not applicable/relevant for your study

no: not applicable, this is a protocol

13b-i) Attrition diagram

Strongly recommended: An attrition diagram (e.g., proportion of participants still logging in or using the intervention/comparator in each group plotted over time, similar to a survival curve) or other figures or tables demonstrating usage/dose/engagement.

	1	2	3	4	5	
subitem not at all important	(•)	( )	( )	( )	( )	essential

Clear selection

Does your paper address subitem 13b-i?

Copy and paste relevant sections from the manuscript or cite the figure number if applicable (include quotes in quotation marks "like this" to indicate direct quotes from your manuscript), or elaborate on this item by providing additional information not in the ms, or briefly explain why the item is not applicable/relevant for your study

no: not applicable, this is a protocol

14a) Dates defining the periods of recruitment and follow-up

Does your paper address CONSORT subitem 14a? *

Copy and paste relevant sections from the manuscript (include quotes in quotation marks "like this" to indicate direct quotes from your manuscript), or elaborate on this item by providing additional information not in the ms, or briefly explain why the item is not applicable/relevant for your study

yes: The Tushirikiane trial launched on February 15, 2020, recruiting a total of 452 participants. Data collection was paused for 8 months owing to COVID-19. Data collection for wave 2 resumed on November 18, 2020, and as of December 10, 2020, a total of 295 participants have been followed up. Data collection for the third, and final, wave will be conducted between February and March 2021. The final follow-up to purchase back unused HIV self-testing kits will occur in June 2021.

14a-i) Indicate if critical “secular events” fell into the study period

Indicate if critical “secular events” fell into the study period, e.g., significant changes in Internet resources available or “changes in computer hardware or Internet delivery resources”

	1	2	3	4	5	
subitem not at all important	○	◉	○	○	○	essential

Clear selection

Does your paper address subitem 14a-i?

Copy and paste relevant sections from the manuscript (include quotes in quotation marks "like this" to indicate direct quotes from your manuscript), or elaborate on this item by providing additional information not in the ms, or briefly explain why the item is not applicable/relevant for your study

yes: we mention COVID-19: The Tushirikiane trial launched on February 15, 2020, recruiting a total of 452 participants. Data collection was paused for 8 months owing to COVID-19. Data collection for wave 2 resumed on November 18, 2020, and as of December 10, 2020, a total of 295 participants have been followed up. Data collection for the third, and final, wave will be conducted between February and March 2021. The final follow-up to purchase back unused HIV self-testing kits will occur in June 2021.

14b) Why the trial ended or was stopped (early)

Does your paper address CONSORT subitem 14b? *

Copy and paste relevant sections from the manuscript (include quotes in quotation marks "like this" to indicate direct quotes from your manuscript), or elaborate on this item by providing additional information not in the ms, or briefly explain why the item is not applicable/relevant for your study

no: not applicable, this is a protocol

15) A table showing baseline demographic and clinical characteristics for each group

NPT: When applicable, a description of care providers (case volume, qualification, expertise, etc.) and centers (volume) in each group

Does your paper address CONSORT subitem 15? *

Copy and paste relevant sections from the manuscript (include quotes in quotation marks "like this" to indicate direct quotes from your manuscript), or elaborate on this item by providing additional information not in the ms, or briefly explain why the item is not applicable/relevant for your study

no: not applicable, this is a protocol

15-i) Report demographics associated with digital divide issues

In ehealth trials it is particularly important to report demographics associated with digital divide issues, such as age, education, gender, social-economic status, computer/Internet/ehealth literacy of the participants, if known.

	1	2	3	4	5	
subitem not at all important	(•)	( )	( )	( )	( )	essential

Clear selection

Does your paper address subitem 15-i? *

Copy and paste relevant sections from the manuscript (include quotes in quotation marks "like this" to indicate direct quotes from your manuscript), or elaborate on this item by providing additional information not in the ms, or briefly explain why the item is not applicable/relevant for your study

no: not applicable, this is a protocol. As per above we will do analyses testing covariates including age and gender.

16) For each group, number of participants (denominator) included in each analysis and whether the analysis was by original assigned groups

16-i) Report multiple “denominators” and provide definitions

Report multiple “denominators” and provide definitions: Report N’s (and effect sizes) “across a range of study participation [and use] thresholds” [1], e.g., N exposed, N consented, N used more than x times, N used more than y weeks, N participants “used” the intervention/comparator at specific pre-defined time points of interest (in absolute and relative numbers per group). Always clearly define “use” of the intervention.

	1	2	3	4	5	
subitem not at all important	(●)	( )	( )	( )	( )	essential

Clear selection

Does your paper address subitem 16-i? *

Copy and paste relevant sections from the manuscript (include quotes in quotation marks "like this" to indicate direct quotes from your manuscript), or elaborate on this item by providing additional information not in the ms, or briefly explain why the item is not applicable/relevant for your study

no: not applicable, this is a protocol

16-ii) Primary analysis should be intent-to-treat

Primary analysis should be intent-to-treat, secondary analyses could include comparing only “users”, with the appropriate caveats that this is no longer a randomized sample (see 18-i).

	1	2	3	4	5	
subitem not at all important	(●)	( )	( )	( )	( )	essential

Clear selection

Does your paper address subitem 16-ii?

Copy and paste relevant sections from the manuscript (include quotes in quotation marks "like this" to indicate direct quotes from your manuscript), or elaborate on this item by providing additional information not in the ms, or briefly explain why the item is not applicable/relevant for your study

Yes: Primary analysis will involve intention-to-treat analysis (data from participants will be analyzed according to their allocation, irrespective of whether they actually received that intervention).

17a) For each primary and secondary outcome, results for each group, and the estimated effect size and its precision (such as 95% confidence interval)

Does your paper address CONSORT subitem 17a? *

Copy and paste relevant sections from the manuscript (include quotes in quotation marks "like this" to indicate direct quotes from your manuscript), or elaborate on this item by providing additional information not in the ms, or briefly explain why the item is not applicable/relevant for your study

no: not applicable, this is a protocol, but we do include this in the methods: Primary analysis will involve intention-to-treat analysis (data from participants will be analyzed according to their allocation, irrespective of whether they actually received that intervention). Between-group comparisons will be performed using multilevel mixed effect logistic or linear regression models (to account for clustering), depending on which outcome is being evaluated. For these models, the intervention group will be entered as a fixed effect. The level of significance will be set at α=.05, but adjusted using the Bonferroni method for secondary outcomes [53]. The results will be expressed as odds ratios or mean differences as appropriate, accompanied by 95% CIs and P values. We will conduct adjusted analysis for the primary outcome (changes in HIV testing frequency) to investigate the role of various covariates in the relative effect. We will build mixed effect multilevel logistic regression models with the intervention group as the independent variable and HIV testing uptake in the past 3 months (yes/no) as the dependent variable. Covariates (eg, age) will be entered as a block. We will explore gender differences in primary and secondary intervention outcomes.

17a-i) Presentation of process outcomes such as metrics of use and intensity of use

In addition to primary/secondary (clinical) outcomes, the presentation of process outcomes such as metrics of use and intensity of use (dose, exposure) and their operational definitions is critical. This does not only refer to metrics of attrition (13-b) (often a binary variable), but also to more continuous exposure metrics such as “average session length”. These must be accompanied by a technical description how a metric like a “session” is defined (e.g., timeout after idle time) [1] (report under item 6a).

	1	2	3	4	5	
subitem not at all important	◉	○	○	○	○	essential

Clear selection

Does your paper address subitem 17a-i?

Copy and paste relevant sections from the manuscript (include quotes in quotation marks "like this" to indicate direct quotes from your manuscript), or elaborate on this item by providing additional information not in the ms, or briefly explain why the item is not applicable/relevant for your study

no: not applicable, this is a protocol

17b) For binary outcomes, presentation of both absolute and relative effect sizes is recommended

Does your paper address CONSORT subitem 17b? *

Copy and paste relevant sections from the manuscript (include quotes in quotation marks "like this" to indicate direct quotes from your manuscript), or elaborate on this item by providing additional information not in the ms, or briefly explain why the item is not applicable/relevant for your study

no: not applicable, this is a protocol

18) Results of any other analyses performed, including subgroup analyses and adjusted analyses, distinguishing pre-specified from exploratory

Does your paper address CONSORT subitem 18? *

Copy and paste relevant sections from the manuscript (include quotes in quotation marks "like this" to indicate direct quotes from your manuscript), or elaborate on this item by providing additional information not in the ms, or briefly explain why the item is not applicable/relevant for your study

no: not applicable, this is a protocol

18-i) Subgroup analysis of comparing only users

A subgroup analysis of comparing only users is not uncommon in ehealth trials, but if done, it must be stressed that this is a self-selected sample and no longer an unbiased sample from a randomized trial (see 16-iii).

	1	2	3	4	5	
subitem not at all important	(•)	( )	( )	( )	( )	essential

Clear selection

Does your paper address subitem 18-i?

Copy and paste relevant sections from the manuscript (include quotes in quotation marks "like this" to indicate direct quotes from your manuscript), or elaborate on this item by providing additional information not in the ms, or briefly explain why the item is not applicable/relevant for your study

no: not applicable, this is a protocol

19) All important harms or unintended effects in each group

(for specific guidance see CONSORT for harms)

Does your paper address CONSORT subitem 19? *

Copy and paste relevant sections from the manuscript (include quotes in quotation marks "like this" to indicate direct quotes from your manuscript), or elaborate on this item by providing additional information not in the ms, or briefly explain why the item is not applicable/relevant for your study

no: not applicable, this is a protocol

19-i) Include privacy breaches, technical problems

Include privacy breaches, technical problems. This does not only include physical “harm” to participants, but also incidents such as perceived or real privacy breaches [1], technical problems, and other unexpected/unintended incidents. “Unintended effects” also includes unintended positive effects [2].

	1	2	3	4	5	
subitem not at all important	(•)	( )	( )	( )	( )	essential

Clear selection

Does your paper address subitem 19-i?

Copy and paste relevant sections from the manuscript (include quotes in quotation marks "like this" to indicate direct quotes from your manuscript), or elaborate on this item by providing additional information not in the ms, or briefly explain why the item is not applicable/relevant for your study

no: not applicable, this is a protocol

19-ii) Include qualitative feedback from participants or observations from staff/researchers

Include qualitative feedback from participants or observations from staff/researchers, if available, on strengths and shortcomings of the application, especially if they point to unintended/unexpected effects or uses. This includes (if available) reasons for why people did or did not use the application as intended by the developers.

	1	2	3	4	5	
subitem not at all important	(•)	( )	( )	( )	( )	essential

Clear selection

Does your paper address subitem 19-ii?

Copy and paste relevant sections from the manuscript (include quotes in quotation marks "like this" to indicate direct quotes from your manuscript), or elaborate on this item by providing additional information not in the ms, or briefly explain why the item is not applicable/relevant for your study

no: not applicable, this is a protocol

DISCUSSION

22) Interpretation consistent with results, balancing benefits and harms, and considering other relevant evidence

NPT: In addition, take into account the choice of the comparator, lack of or partial blinding, and unequal expertise of care providers or centers in each group

22-i) Restate study questions and summarize the answers suggested by the data, starting with primary outcomes and process outcomes (use)

Restate study questions and summarize the answers suggested by the data, starting with primary outcomes and process outcomes (use).

	1	2	3	4	5	
subitem not at all important	◉	○	○	○	○	essential

Clear selection

Does your paper address subitem 22-i? *

Copy and paste relevant sections from the manuscript (include quotes in quotation marks "like this" to indicate direct quotes from your manuscript), or elaborate on this item by providing additional information not in the ms, or briefly explain why the item is not applicable/relevant for your study

no: not applicable, this is a protocol

22-ii) Highlight unanswered new questions, suggest future research

Highlight unanswered new questions, suggest future research.

	1	2	3	4	5	
subitem not at all important	◉	○	○	○	○	essential

Clear selection

Does your paper address subitem 22-ii?

Copy and paste relevant sections from the manuscript (include quotes in quotation marks "like this" to indicate direct quotes from your manuscript), or elaborate on this item by providing additional information not in the ms, or briefly explain why the item is not applicable/relevant for your study

no: not applicable, this is a protocol

20) Trial limitations, addressing sources of potential bias, imprecision, and, if relevant, multiplicity of analyses

20-i) Typical limitations in ehealth trials

Typical limitations in ehealth trials: Participants in ehealth trials are rarely blinded. Ehealth trials often look at a multiplicity of outcomes, increasing risk for a Type I error. Discuss biases due to non-use of the intervention/usability issues, biases through informed consent procedures, unexpected events.

	1	2	3	4	5	
subitem not at all important	○	◉	○	○	○	essential

Clear selection

Does your paper address subitem 20-i? *

Copy and paste relevant sections from the manuscript (include quotes in quotation marks "like this" to indicate direct quotes from your manuscript), or elaborate on this item by providing additional information not in the ms, or briefly explain why the item is not applicable/relevant for your study

yes: The primary study limitations are loss to follow-up, missing data points, and study delays due to COVID-19.

21) Generalisability (external validity, applicability) of the trial findings

NPT: External validity of the trial findings according to the intervention, comparators, patients, and care providers or centers involved in the trial

21-i) Generalizability to other populations

Generalizability to other populations: In particular, discuss generalizability to a general Internet population, outside of a RCT setting, and general patient population, including applicability of the study results for other organizations

	1	2	3	4	5	
subitem not at all important	○	◉	○	○	○	essential

Clear selection

Does your paper address subitem 21-i?

Copy and paste relevant sections from the manuscript (include quotes in quotation marks "like this" to indicate direct quotes from your manuscript), or elaborate on this item by providing additional information not in the ms, or briefly explain why the item is not applicable/relevant for your study

Our research will provide gender- and age-stratified analyses, as well as an understanding of the potential added benefits of SMS support strategies alongside HIV self-testing to inform differentiated HIV testing strategies among urban refugee and displaced youth, which can be adapted for diverse contexts.

21-ii) Discuss if there were elements in the RCT that would be different in a routine application setting

Discuss if there were elements in the RCT that would be different in a routine application setting (e.g., prompts/reminders, more human involvement, training sessions or other co-interventions) and what impact the omission of these elements could have on use, adoption, or outcomes if the intervention is applied outside of a RCT setting.

	1	2	3	4	5	
subitem not at all important	(•)	( )	( )	( )	( )	essential

Clear selection

Does your paper address subitem 21-ii?

Copy and paste relevant sections from the manuscript (include quotes in quotation marks "like this" to indicate direct quotes from your manuscript), or elaborate on this item by providing additional information not in the ms, or briefly explain why the item is not applicable/relevant for your study

No: not applicable as this is a clinical based intervention

OTHER INFORMATION

23) Registration number and name of trial registry

Does your paper address CONSORT subitem 23? *

Copy and paste relevant sections from the manuscript (include quotes in quotation marks "like this" to indicate direct quotes from your manuscript), or elaborate on this item by providing additional information not in the ms, or briefly explain why the item is not applicable/relevant for your study

yes: ClinicalTrials.gov NCT04504097;
https://clinicaltrials.gov/ct2/show/NCT04504097.

24) Where the full trial protocol can be accessed, if available

Does your paper address CONSORT subitem 24? *

Cite a Multimedia Appendix, other reference, or copy and paste relevant sections from the manuscript (include quotes in quotation marks "like this" to indicate direct quotes from your manuscript), or elaborate on this item by providing additional information not in the ms, or briefly explain why the item is not applicable/relevant for your study

no because this is the full trial protocol

25) Sources of funding and other support (such as supply of drugs), role of funders

Does your paper address CONSORT subitem 25? *

Copy and paste relevant sections from the manuscript (include quotes in quotation marks "like this" to indicate direct quotes from your manuscript), or elaborate on this item by providing additional information not in the ms, or briefly explain why the item is not applicable/relevant for your study

yes: This study has been funded by the Canadian Institutes of Health Research (project grant 389142). Funding agencies played no role in the design or execution of the study. CL is also funded by the Canada Research Chairs program (Tier 2: Logie), Canada Foundation for Innovation (Logie's SSHINE Lab), and the Ontario Ministry of Research and Innovation (ERA: Logie).

X27) Conflicts of Interest (not a CONSORT item)

X27-i) State the relation of the study team towards the system being evaluated

In addition to the usual declaration of interests (financial or otherwise), also state the relation of the study team towards the system being evaluated, i.e., state if the authors/evaluators are distinct from or identical with the developers/sponsors of the intervention.

	1	2	3	4	5	
subitem not at all important	○	◉	○	○	○	essential

Clear selection

Does your paper address subitem X27-i?

Copy and paste relevant sections from the manuscript (include quotes in quotation marks "like this" to indicate direct quotes from your manuscript), or elaborate on this item by providing additional information not in the ms, or briefly explain why the item is not applicable/relevant for your study

yes

About the CONSORT EHEALTH checklist

As a result of using this checklist, did you make changes in your manuscript? *

- ( ) yes, major changes
- ( ) yes, minor changes
- (•) no

What were the most important changes you made as a result of using this checklist?

none (it is a protocol paper that is in press)

How much time did you spend on going through the checklist INCLUDING making changes in your manuscript *

2 hours

As a result of using this checklist, do you think your manuscript has improved? *

- ○ yes
- ◉ no
- ○ Other:

Would you like to become involved in the CONSORT EHEALTH group?

This would involve for example becoming involved in participating in a workshop and writing an "Explanation and Elaboration" document

- ○ yes
- ◉ no
- ○ Other:

Clear selection

Any other comments or questions on CONSORT EHEALTH

1. This is not relevant for a protocol paper, half of these questions were irrelevant.
2. We had already submitted the completed consort form, and our article is in press, so this was a redundant process. Also, most of these questions are irrelevant so better to have a word document such as the consort is in, as easier to fill in.

STOP - Save this form as PDF before you click submit

To generate a record that you filled in this form, we recommend to generate a PDF of this page (on a Mac, simply select "print" and then select "print as PDF") before you submit it.

When you submit your (revised) paper to JMIR, please upload the PDF as supplementary file.

Don't worry if some text in the textboxes is cut off, as we still have the complete information in our database. Thank you!

Final step: Click submit !

Click submit so we have your answers in our database!

Submit

Never submit passwords through Google Forms.

This content is neither created nor endorsed by Google. Report Abuse - Terms of Service - Privacy Policy

Google Forms
