# Supplementary material for: Mobile Health–Supported HIV Self-Testing Strategy Among Urban Refugee and Displaced Youth in Kampala, Uganda: Protocol for a Cluster Randomized Trial (Tushirikiane, Supporting Each Other)
Source: JMIR Res Protoc. 2021 Feb 2;10(2):e26192. doi: 10.2196/26192 (PMC7886618; doi:10.2196/26192)
Supplement: Multimedia Appendix 2 [file resprot_v10i2e26192_app2.pdf]

**Review Type / Type d'évaluation:**	Reviewer 1 / Évaluateur 1
**Name of Applicant / Nom du chercheur:**	Logie, Carmen
**Application No. / Numéro de demande:**	389142
**Agency / Agence:**	CIHR/IRSC
**Competition / Concours:**	Project Grant/Subvention Projet
**Committee / Comité:**	Psychosocial, Socio-cultural & Behavioural Determinants of Health/Déterminants psychosociaux, socio-culturels et comportementaux de la santé
**Title / Titre:**	Tushirikiane (Supporting Each Other): Development, Implementation and Evaluation of Novel HIV Self-Testing Delivery Approaches with Urban Displaced and Refugee Adolescent Girls in Uganda

Summary of Application/Résumé de la demande:

The proposed project addresses an UNAIDS mandate to increase persons knowing their HIV status to 90% by 2020. The goal of the project is to develop evidence-based, effective strategies to increase HIV self-testing uptake and linkage to HIV care among displaced/refugee adolescent girls in Uganda, Sub-Saharan Africa and humanitarian contexts. The team proposes to collaborate with displaced/refugee adolescent girls, government, and community agencies to: 1) develop an m-health intervention to increase HIV Self-Testing (HIVST) uptake; 2) implement and evaluate the effectiveness of HIVST in isolation or in combination with an m-health intervention to achieve routine (every 3 month) HIV testing and linkage to care; 3) apply participatory knowledge mobilization to facilitate HIVST scale-up.

Specifically, the team proposes a mixed-methods approach with 3 phases. Phase 1 (qualitative) involves working with a youth advisory board and refugee/displaced peer navigators. The team proposes to conduct 6 focus groups (n=60) and 12 key informant interviews to develop an m-health intervention (healthcare delivered on mobile-phones: supportive text messages, WhatsApp discussions). In Phase 2 (quantitative) they will implement a 3-arm cluster randomized trial with refugee/displaced adolescent girls aged 15-19 (n=432) in 3 slums (clusters) in Kampala. Clusters will be randomized to 1 of 3 arms: 1) HIVST; 2) HIVST + m-health; and 3) standard of care (clinic based HIV testing). They will conduct surveys (baseline, 3-month, 6-month follow-up) to assess changes in HIV testing, HIV status knowledge, and linkage to HIV care. Phase 3 involves a process evaluation and participatory knowledge mobilization.

**Review Type / Type d'évaluation:**	Reviewer 1 / Évaluateur 1
**Name of Applicant / Nom du chercheur:**	Logie, Carmen
**Application No. / Numéro de demande:**	389142
**Agency / Agence:**	CIHR/IRSC
**Competition / Concours:**	Project Grant/Subvention Projet
**Committee / Comité:**	Psychosocial, Socio-cultural & Behavioural Determinants of Health/Déterminants psychosociaux, socio-culturels et comportementaux de la santé
**Title / Titre:**	Tushirikiane (Supporting Each Other): Development, Implementation and Evaluation of Novel HIV Self-Testing Delivery Approaches with Urban Displaced and Refugee Adolescent Girls in Uganda

Strengths and Weaknesses/Forces et faiblesses:

The proposal addresses a World Health Organization adolescent HIV research priority to identify strategies/interventions to improve access to, and uptake of, HIV testing services, including HIV-self testing (HIVST). As the team points out, empirical evidence is needed immediately to advance HIV prevention and care, particularly with Ugandan urban displaced/refugee adolescent girls, a group at elevated risk. It is highly innovative to examine how m-health (healthcare delivered on mobile-phones), congruent with how adolescents learn and socialize, can improve linkage to care with HIVST. The rationale of the project idea is superb. The overall goals and objectives are well-defined and important. The project seems likely to advance both knowledge of HIV testing and to lead to the development of an evidence-based intervention to increase the use of self-tests for HIV in vulnerable populations.

The proposed mixed method, multi-stage approach seems particularly appropriate to this project. Starting with qualitative methods will increase the probability that the design of the intervention will be culturally sensitive. The RCT appears well designed and feasible.

Timelines appear to be realistic.

The proposed study is important for increasing understanding of HIV self-testing among adolescent girls in Sub-Saharan Africa. As the team points out, despite three-fold higher rates of HIV among adolescent girls vs. adolescent boys in Uganda, there has been limited attention to effective, culturally and gender specific HIV prevention strategies, including HIV self testing and linkage to HIV care. Importantly, the work aligns with Canada’s 2017 Feminist International Assistance Policy to advance gender equality.

The team is cross-national and multidisciplinary (Social Work, Nursing, Medicine, Biostatistics, Medical Anthropology), which is very important for the proposed study given the topic and population to be studied. Included is a knowledge user representative from the Ugandan Ministry of Health.

The applicant has a track record in publishing and success in obtaining grants for her research.

The budget appears realistic and well-justified, and it is recommended that it be accepted as described.

**Review Type / Type d'évaluation:**	Reviewer 2 / Évaluateur 2
**Name of Applicant / Nom du chercheur:**	Logie, Carmen
**Application No. / Numéro de demande:**	389142
**Agency / Agence:**	CIHR/IRSC
**Competition / Concours:**	Project Grant/Subvention Projet
**Committee / Comité:**	Psychosocial, Socio-cultural & Behavioural Determinants of Health/Déterminants psychosociaux, socio-culturels et comportementaux de la santé
**Title / Titre:**	Tushirikiane (Supporting Each Other): Development, Implementation and Evaluation of Novel HIV Self-Testing Delivery Approaches with Urban Displaced and Refugee Adolescent Girls in Uganda

Summary of Application/Résumé de la demande:

HIV is the main cause of death for adolescents aged 10-19 years in Sub-Saharan Africa. Specifically, although it is more prevalent in girls in Uganda, half of adolescent girls in this country do not know their HIV status, with refugees and displaced girls being the most neglected. HIV self-testing (HIVST) is a promising approach to reach such neglected populations. The proposed research is a three-arm cluster randomized controlled trial comparing a novel, contextually tailored, m-health HIVST delivery strategy to increase routine HIV testing uptake in adolescent girls in Uganda, to regular HIVST and to routine care (clinic HIV testing).

The PI is an Assistant Professor of Social Work at the University of Toronto. She currently holds grants from the Ontario Ministry of Research and Innovation as well as SSHRC and CIHR, and received a CFI grant for her SSHINE Lab. She lists 76 published papers, two edited books and one book chapter since 2010. The large multidisciplinary team holds expertise in relation to the proposed study.

**Review Type / Type d'évaluation:**	Reviewer 2 / Évaluateur 2
**Name of Applicant / Nom du chercheur:**	Logie, Carmen
**Application No. / Numéro de demande:**	389142
**Agency / Agence:**	CIHR/IRSC
**Competition / Concours:**	Project Grant/Subvention Projet
**Committee / Comité:**	Psychosocial, Socio-cultural & Behavioural Determinants of Health/Déterminants psychosociaux, socio-culturels et comportementaux de la santé
**Title / Titre:**	Tushirikiane (Supporting Each Other): Development, Implementation and Evaluation of Novel HIV Self-Testing Delivery Approaches with Urban Displaced and Refugee Adolescent Girls in Uganda

Strengths and Weaknesses/Forces et faiblesses:

Strengths

The need for, and rationale underlying the proposed study are compelling. HIVST among youth has been shown to be feasible. Relevant letters of support are included. The proposal is well structured and covers a wealth of background literature supporting the relevance of the proposed three-phase research. Study methods have been used with other populations (e.g., First Nations in Canada).

Weaknesses

Many listed publications were submissions. Publications are in journals of varying quality.

The proposal is overly dense, contains many acronyms, etc., making it difficult to follow at times.

Although there is a wealth of information on many aspects of the contextual elements integral to the proposed study, the proportion of adolescent girls in Uganda who own a cell phone is not mentioned. This is crucial to the feasibility of the proposed study. The fact that there is free evening Internet in Kampala does not address this issue. This also relates to future implementation: if penetration of the Internet in Uganda is 31.3%, how can the new intervention be sustained?

The neurodevelopment argument of sexual risk taking is of limited scope as most adolescents do not engage in sexual risk taking and other psychosocial developmental factors tend to drive sexual risk behaviour.

How will parental consent be obtained for youth younger than 18? I understand that Uganda does not require parental consent for HIV testing for persons aged 12 and over, but what about consent for participating in clinical research that involves receiving an intervention?

How feasible is it for peer navigators to be in charge of data collection? Has this method been used before and does it yield high quality data?

Feasibility and rate of recruitment of youth for the cRCT is not clear. Piloting this aspect of the study would be beneficial and ensure proprer planning and justification of the timeline for the proposed RCT. Pilot data are also missing to support successful participant retention in this phase of the study.

Investigators purport that they can link the survey ID to resources accessed but it is not clear how this will be achieved.

Twenty-four girls per group discussion seems excessive and not conducive to participation.

**Review Type / Type d'évaluation:**	Reviewer 3 / Évaluateur 3
**Name of Applicant / Nom du chercheur:**	Logie, Carmen
**Application No. / Numéro de demande:**	389142
**Agency / Agence:**	CIHR/IRSC
**Competition / Concours:**	Project Grant/Subvention Projet
**Committee / Comité:**	Psychosocial, Socio-cultural & Behavioural Determinants of Health/Déterminants psychosociaux, socio-culturels et comportementaux de la santé
**Title / Titre:**	Tushirikiane (Supporting Each Other): Development, Implementation and Evaluation of Novel HIV Self-Testing Delivery Approaches with Urban Displaced and Refugee Adolescent Girls in Uganda

Summary of Application/Résumé de la demande:

The overall goal of the project is to develop evidence-based, effective strategies to increase HIV self-testing uptake and linkage to HIV care among displaced/refugee adolescent girls in Uganda, SSA and humanitarian contexts. The specific objectives are to: 1. Develop an m-health intervention (WhatsApp discussions, two-way supportive text messages) to increase routine HIVST uptake, HIV status knowledge and HIV care with refugee and displaced adolescent girls in Uganda; 2. Evaluate the feasibility and effectiveness of: HIVST alone, and HIVST in combination with m-health, in increasing routine HIV testing, HIV status knowledge, and linkage to confirmatory testing and HIV care. and 3. Mobilise knowledge of implementation processes and effectiveness of HIVST delivery approaches.

The mixed methods project will be conducted in three phases.
Phase 1 (qualitative): Working closely with a youth advisory board and peer navigators, the researchers will conduct 6 focus groups (n=60) and 12 key informant interviews to inform m-health intervention development (supportive text messages, WhatsApp discussions) grounded in the Behaviour Change Wheel. Phase 2 (quantitative): A 3-arm cluster randomized trial with refugee/displaced adolescent girls aged 15-19 (n=432) in 3 slums (clusters) in Kampala. Clusters will be randomized to 1 of 3 arms: 1) HIVST; 2) HIVST + m-health; and 3) standard of care. The researchers will conduct surveys (baseline, 3-month, 6-month follow-up) to assess changes in HIV testing, HIV status knowledge, and linkage to HIV care. Phase 3 (qualitative): Process evaluation and participatory knowledge mobilization.

The research team includes a combination of multi-disciplinary, international group of researchers and knowledge users.

**Review Type / Type d'évaluation:**	Reviewer 3 / Évaluateur 3
**Name of Applicant / Nom du chercheur:**	Logie, Carmen
**Application No. / Numéro de demande:**	389142
**Agency / Agence:**	CIHR/IRSC
**Competition / Concours:**	Project Grant/Subvention Projet
**Committee / Comité:**	Psychosocial, Socio-cultural & Behavioural Determinants of Health/Déterminants psychosociaux, socio-culturels et comportementaux de la santé
**Title / Titre:**	Tushirikiane (Supporting Each Other): Development, Implementation and Evaluation of Novel HIV Self-Testing Delivery Approaches with Urban Displaced and Refugee Adolescent Girls in Uganda

Strengths and Weaknesses/Forces et faiblesses:

The proposal addresses a significant and urgent health need of a vulnerable population (HIV among displaced and refugee adolescent girls in Uganda). The researchers make a compelling case for the importance of the research, including a detailed analysis of the HIV risk of the target group, the value of HIV self-testing and the use of mobile health approach.

The mixed methods approach effectively integrates both qualitative and quantitative approaches to program design and evaluation. The initial year of development work, with meaningful engagement of the community (forming an advisory team, training peer navigators, conducting youth focus groups and key informant interviews) will be integral to understanding the individual issues and the broader social context in order to design an acceptable, feasible, culturally relevant approach to empower girls to engage in HIV self-testing. The clustered RCT approach makes sense given the social context of the slums and there was a good description of the intervention and analysis approaches. Sample size justification was provided, although a 3-4 month recruitment period for 144 participants and 10% drop-out rate is quite ambitious given the many complex challenges faced by the displaced young women. It will be interesting to see if the strategy of maintaining weekly contact is effective in maintaining engagement. It will be important to be clear about how responses to problems identified in these check-ins will be handled, since this may add an additional layer of complexity to the role of those who are responding to the text messages. It was good to see that the study incorporates not only self-testing, but also promotes linkages to additional community supports for HIV intervention. The researchers have done a good job of considering how to manage social desirability bias. The effectiveness implementation hybrid design to simultaneously collect data on the intervention and the implementation process will yield important implementation data that will inform interpretation of the outcomes and recommendations for implementation and evaluation. The proposal integrated a clear sex/gender based framework throughout the study, including theoretical and scientific understanding of gender issues, approach to data collection, analysis and knowledge translation. The researchers have also done a good job of considering how to track process as well as outcome data. Since participation in the intervention is over a 6 month period, are there plans to ensure sustainability once the project is complete? Since you will have invested a lot of time in training and establishing processes of implementation, it will be important to ensure that the work continues beyond the data collection period.

This is an ambitious, but important project that has the potential to make a significant difference in the lives of a group of vulnerable young women. The proposed intervention could have a significant impact on the community since HIV testing can be a key entry point for HIV prevention and antiretroviral therapy—preventing onward HIV transmission. The research team has established a clear foundation of support from the community with letters of support from 10 Uganda-based organisations, including high level decision makers as well as front-line champions. The primary investigator has done similar research in Haiti, therefore will be able to transfer some of this knowledge to this research in Uganda. As noted in the proposal, the project will build on the team's CIHR Planning Grant and collective expertise in global HIV research with adolescents, displaced people, and m-health. Overall, the proposal was thoughtfully prepared and well-written.
